# Effect of the double bond conjugation on the vascular physiology and nitric oxide production of isomers of eicosapentaenoic and docosahexaenoic acids prepared from shark oil

**DOI:** 10.1371/journal.pone.0229435

**Published:** 2020-02-27

**Authors:** Carmen Gonzalez, Ana Sonia Silva-Ramirez, Gabriela Navarro-Tovar, Juan Jesus Barrios-Capuchino, Alejandro Rocha-Uribe

**Affiliations:** 1 Facultad de Ciencias Quimicas, Universidad Autonoma de San Luis Potosi, San Luis Potosí, San Luis Potosi, Mexico; 2 Centro de Investigacion en Ciencias de la Salud y Biomedicina (CICSaB), Universidad Autonoma de San Luis Potosi, San Luis Potosi, San Luis Potosi, Mexico; 3 Consejo Nacional de Ciencia y Tecnologia, Mexico City, Mexico; University of Rochester, UNITED STATES

## Abstract

A collection of evidence suggests that conjugation of double bonds of eicosapentaenoic (EPA) and docosahexaenoic (DHA) acids, omega-3 polyunsaturated fatty acids (n-3 PUFAs), increases their anticarcinogenic activity; however, the effect of such conjugation on vascular tone activity remains unknown. We propose that the mixture of conjugated PUFAs exerts higher vasorelaxation activity than the corresponding mixture of nonconjugated PUFAs. The vascular response to different concentrations of conjugated and nonconjugated isomers of EPA and DHA, among other fatty acids (FAs) naturally present in shark oil, and the role of nitric oxide (NO) as a vasorelaxant agent were investigated. Both conjugated EPA (CEPA) and conjugated DHA (CDHA) were prepared by alkaline isomerization of all PUFAs contained in shark oil. Different concentrations of conjugated and nonconjugated PUFAs were placed in contact with precontracted aortic rings of Wistar rats to assess their effect on vascular tone. All tested samples exerted a vasorelaxant effect. Compared to nonconjugated PUFAs, conjugated isomers exhibited an increase in the dilatation of the aortic rings (P<0.001) in a dose-dependent manner (P<0.001). In addition, nonconjugated PUFAs produced nitric oxide (NO) in a dose-dependent manner, while conjugated PUFAs did not, suggesting that their dilatation mechanism is not totally dependent on NO.

## Introduction

The dietary intake of large amounts of n-3 PUFAs, such as EPA and DHA, which are mainly contained in marine oils (i.e., fish, whale and seaweed), has been linked to a low risk of cardiovascular disease [1]–[7]. The results collected by several basic studies suggest that PUFAs significantly improve cardiovascular disease mechanisms in both tissues and cells. For instance, 1) n-3 PUFAs reduce endothelial dysfunction through a modification of eicosanoid biosynthesis [8]–[10] and by regulating the levels of nitric oxide (NO) [11]–[13]; 2) n-3 PUFAs also modify the levels of lipids by activating transcription factors and mechanisms such as beta oxidation [14]–[16]; and 3) n-3 PUFAs participate in the signaling pathway of the interleukin-2 receptor, interleukin-1 receptor and other anti-inflammatory mechanisms [17]–[19].

However, the evidence in clinical studies is controversial, as a random-effect meta-analysis of the study of n-3 PUFAs showed in 2018 [5]. Epidemiological studies as well as randomized controlled trials have demonstrated that a high intake of long-chain fatty acids or PUFAs, such as eicosapentaenoic acid (EPA) and docosahexaenoic acid (DHA), reduces the risk of cardiovascular disease (CVD). Although a reduction in the risk of CVD has not been related to high EPA and DHA intake in healthy subjects, significantly lower levels of very-low-density lipoproteins have been linked to high DHA doses. In this respect, PUFAs have been associated with a reduction in serum triglycerides through two paths: 1) a partial depletion of the hepatic synthesis of very-low-density lipoproteins and 2) by enhancing fatty acid catabolism and accelerating triglyceride clearance from the plasma [7]. In 2017, Siscovick et al., in a science advisory of the American Heart Association (AHA), collected multiple randomized controlled trials where the supplementary administration of fish oil (rich in DHA and EPA) to patients with CVD was used. Those authors concluded that a modest reduction in mortality (10%) justifies the use of fish oil in this population. However, such authors do not recommend the use of fish oil in patients with heart failure, diabetes and prediabetes since evidence did not demonstrate a reduction in mortality [6]. Furthermore, other clinical studies report that fish oil supplements could improve blood flow by arterial dilation and, consequently, heart mechanical function [7], [20]. More recently, Skulas-Ray et al. [21] reported a science advisory from the AHA collecting the available clinical evidence related to the effect of n-3 PUFAs on the plasma concentration of triglycerides and other lipids in individuals with elevated triglycerides [21]. Considering that DHA+EPA (4 g/day total) or EPA alone has been used since 2002 for the treatment of very high triglycerides (≥ 500 mg/dL in plasma), the observations collected by those authors include the following: 1) EPA+DHA reduce triglycerides by ≥ 30% with concurrent increases in low-density lipoprotein cholesterol, while EPA alone doses do not increase low-density lipoprotein cholesterol; 2) n-3 PUFAs decrease non–high-density lipoprotein cholesterol and apolipoprotein B; and 3) n-3 PUFAs achieve a 25% reduction in major adverse cardiovascular events in REDUCE-IT (Reduction of Cardiovascular Events With EPA Intervention Trial). Thus, the scientific advisory for the AHA concludes that prescription of EPA+DHA or EPA alone (4 g/day) could be an effective therapy or coadjuvant in pharmacological therapy for individuals with very high triglycerides [21].

It has been reported that n-3 PUFAs could increase the bioavailability of NO levels and therefore induce vasodilation [22]. However, some other mechanisms not related to NO have been proposed for the vasodilation effect exerted by n-3 PUFAs in rats; for instance, Engler et al. [23] showed that EPA exerts an endothelium- and nitric oxide-independent vasorelaxant effect in WKY rat aortae through the production of prostanoids that activate K^+^_ATP_ channels. Inhibition of Ca^2+^ mobilization from intracellular pools and influx through the non-L-type, but not the L-type, Ca^2+^ channels are also possible mechanisms of action of EPA, since the relaxant effect of EPA was significantly inhibited by indomethacin, a cyclo-oxygenase inhibitor, but not by a nitric oxide (NO) synthesis blocker (NO-nitro-L-arginine methyl ester hydrochloride: L-NAME) [23]. In addition, the removal of the endothelium did not alter EPA-induced relaxations. Moreover, in Ca^2+^-free and EGTA solutions, EPA significantly inhibited NA-sustained contractions, and the vasorelaxant effects of EPA on NA-induced contractions were significantly inhibited by the K^+^ channel blocker glibenclamide but not tetraethylammonium. Moreover, indomethacin and glibenclamide combined significantly inhibited EPA-induced responses. In conclusion, the authors of that work suggest that the vasorelaxant actions of EPA, at least in this model, are not dependent on nitric oxide and that they are attributed to the primary mechanism of EPA-induced relaxation. In WKY rat aortae, it appears to be mediated by prostanoids that activate K^+^_ATP_ channels and are associated with specific inhibition of Ca^2+^ mobilization from the sarcoplasmic reticulum or other intracellular Ca^2+^ pools, as well as non-L-type Ca^2+^ entry. L-type Ca^2+^ channels are less likely to be involved in EPA-induced relaxation in WKY aortae [23].

On the other hand, n-3 PUFAs compete with the arachidonic acid pathway. Arachidonic acid (AA) is a fatty acid released from cell membrane phospholipids by the increased activity of phospholipase A_2_ (PLA_2_) and, as a consequence, the stimulation of platelets and endothelial cells. The release of arachidonic acid is associated with cyclooxygenase-2 (COX_2_) followed by an increase in thromboxane A_2_ (TXA_2_), a potent promoter of platelet aggregation, and prostacyclin I_2_ (PGI_2_), a potent inhibitor of platelet aggregation [24].

Dong et al. [25] studied the mechanisms in human huPGH-1 cells, where the cell culture medium was enriched with fish oil fatty acids. The results suggested that fish oil-enriched diets disfavor AA oxygenation by altering the composition of the total fatty acid in which huPGHS-1 functions. According to enzyme evaluations, n-3 PUFAs are associated with the production of COX_2_ metabolites (series-3 PGs) and CYP450 metabolites (epoxyeicosatetraenoic acids and epoxydocosapentaenoic acids), which are all involved in vasodilation [25].

Recently, EPA and DHA pretreatments have been associated with a reduction in the vascular tone of the human saphenous vein under both normal and inflammatory conditions [26]. Other findings in animals [27] and humans [28], [29] indicate that DHA plays a more important role than EPA in endothelial regulation.

Limbu et al. [30] evaluated the vasodilation effect of EPA and DHA pure fatty acids on isolated aortic and mesenteric arteries of rats. The NO-independent effects on vasodilation showed a minor role of the endothelium. In addition, COX was not involved, and CYP450 expressed a small effect [30]. Sato et al. [31] related vasodilation in the rat mesenteric artery by inhibiting prostanoid receptor-mediated constrictions when exposed to EPA and DHA [31]. Wiest et al. [32] conducted a mouse model of cigarette smoke-induced endothelial dysfunction and investigated the mechanisms involved in n-3 PUFA vasoprotection. An EPA- and DHA-enriched diet restored flow-mediated dilation by NO-dependent dilation and NO-independent dilation and decreased the levels of both 8-epiprostaglandin-F2α and heme oxygenase-1 mRNA (markers of oxidative stress) [32].

Thus, the heterogeneity in the results on the vasodilation effects triggered by n-PUFAs, which happen due to several factors (i.e., vascular bed type, pretreatment of bed and of PUFAs, etc.), require more studies that should consider both mixtures and isolated n-PUFAs.

Independent of the vasorelaxant effect exerted by n-3 PUFAs, several studies revealed that conjugation of the double bonds of EPA and DHA by alkaline isomerization is associated with an increment in the anticarcinogenic activity in human cancer cell lines of the breast [33], [34], and colon [35], [36], and it has been related to a significant *in vivo* antitumor effect [34], [37], [38].

Thus, we expect a similar effect of conjugation of double bonds of PUFAs. To our knowledge, there are no reports to date that show the effect of conjugated n-3 PUFAs on the regulation of vascular tone.

An extra relevance of this research work is that in most of the aforementioned *ex vivo* studies, these FAs are generally considered separately, and their potential interactions have not been taken into account. Here, we studied the effect of mixtures of FAs as is present in shark oil (conjugated and nonconjugated) on the vascular tone in an *in vitro* system, instead of separating and purifying the specific FAs.

Additionally, the fishing of pelagic and coastal shark species (i.e., *Carcharhinus falciformis*) is an important economic activity in Mexico. However, there is a lack of information about the liver oil composition and its properties from the different shark species in this country. For example, the fatty acid composition of the liver oil of *Carcharhinus falciformis* (from the Caribbean and Mexican waters) has been reported [39]. Such oils were particularly high in n-3 PUFAs, mainly DHA and EPA, depending on the place where the shark was caught. These oil compositional characteristics and their physiological or nutritional properties could be of interest to more efficiently promote the use of the liver (as a source of oil), which is traditionally a byproduct (or waste) of the underdeveloped shark fishery in Mexico. The generation of new industries (i.e., for extraction and processing of such marine oil) would enhance the economic conditions in the region where such activity is practiced.

In the present study, we hypothesized that a mixture of FAs containing conjugated PUFAs present in isomerized shark oil (from *Carcharhinus falciformis*) induces a higher vasorelaxation effect than the corresponding mixture of FAs containing nonconjugated PUFAs (as they are found naturally in the fish oil) due to the conjugation of double bonds of the PUFAs present in the oil.

Accordingly, the effect of the conjugation of PUFAs, mainly EPA and DHA, among other fatty acids contained in shark oil on the vascular response of rat aortic rings was evaluated, which could be relevant for the prevention of cardiovascular diseases. Additionally, the effect of conjugated PUFAs on NO production and their possible association with vasorelaxation were investigated.

## Materials and methods

### Materials

Refined shark oil (from the liver of *Carcharhinus falciformis*, caught in the Gulf of Mexico, Coastal area of Veracruz, 20°21´ N, 95°06´ W) was kindly donated by G. Navarro-Garcia from the Center for Research in Food and Development (Hermosillo, Sonora, Mexico). The oil presented a total PUFA content of 39.5% (where n-3, EPA and DHA constituted 10.3% and 18.6%, respectively). The reagents for the physiologic solution [NaCl, KCl, MgSO_4_, KH_2_PO_4_, CaCl_2_, indomethacin, glucose, HEPES and dimethyl sulfoxide (DMSO)] were provided by Mallinckrodt Baker (Mexico State, Mexico). Phenylephrine (Phe), acetylcholine (ACh) and Supelco 37 Component FAME Mix (Cat. No. 47885-U) were obtained from Sigma-Aldrich Química S.A. de C.V. (Toluca, Estado de Mexico, Mexico). All reagents used for the isomerization reaction were purchased from Fermont (Monterrey Chemicals Products, Monterrey, Nuevo Leon, Mexico).

### Methods

#### Preparation of isomerized shark oil (ISO) and hydrolyzed shark oil (HSO)

ISO [mixture of free fatty acids (FFAs) containing conjugated PUFAs] was prepared by alkaline isomerization of shark oil [34], [36], [40]. Briefly, 400 mg of oil was reacted with 40 mL of KOH-ethylene glycol solution (21:79, w/w) under nitrogen atmosphere for 5 min at 160°C. The resulting product consisted of a mixture of nonesterified FA potassium salts in which the PUFAs (mainly EPA and DHA) were converted to the corresponding conjugated isomers, conjugated EPA or CEPA and conjugated DHA or CDHA, respectively.

HSO (mixture of FFAs containing nonconjugated PUFAs) was obtained by applying the American Oil Chemists’ Society (AOCS) official method Cd 3–25 [41]. Briefly, 800 mg of shark oil was hydrolyzed with 8 mL of KOH-MeOH (4:96, w/v) for 5 min at constant boiling. The resulting product consisted of a mixture of nonesterified FA potassium salts in which the PUFAs (mainly EPA and DHA) were nonconjugated.

The mixtures of nonesterified FA potassium salts that resulted from isomerization and hydrolysis procedures were treated as previously described to recover FAs as FFAs [34], [36], [40]. Soaps (i.e., K salts of FAs) that resulted from the alkaline isomerization reaction were cooled to room temperature, and 40 mL of methanol was added. Then, the mixture was acidified to pH under 2 using a 6 N HCl solution to produce the corresponding FFAs by precipitation of KCl salts. After dilution with 8 mL of distilled water, the lipid fraction (i.e., FFA mixture) was extracted with 20 mL of hexane. The hexane extract was washed with 12 mL of methanol-distilled water (30:70, v/v) and with 12 mL of distilled water and then dried with 5% w/w MgSO_4_. Finally, the FFA mixture containing conjugated or isomerized PUFAs (ISOs) was separated by evaporation of hexane under a nitrogen gas stream. On the other hand, the nonisomerized FFA mixture, HSO (with nonconjugated PUFAs) that resulted from the hydrolysis reaction of shark oil, was extracted in the same way as the above detailed procedure, but with the adjustment in the proportion of reagents according to the amount of oil used. Isomerized and hydrolyzed mixtures of FFAs (ISO and HSO) were stored at -20°C after being purged with nitrogen gas until further analysis and other studies were conducted.

HSO was used as a reference to evaluate the effect of conjugation of double bonds present in the n-3 PUFAs of ISO on the vasorelaxation effect since the only difference in the FA profile between both samples was that PUFAs in ISO were conjugated, while n-3 PUFAs in HSO do not contain conjugated double bonds.

A UV-Vis spectrophotometric analysis of ISO and HSO samples was performed with a Shimadzu UV-160U spectrophotometer (Shimadzu, Kyoto, Japan) to measure the content of conjugated isomers (i.e., dienes, trienes, tetraenes and pentaenes) [40].

#### Fatty acid composition

The FA composition of both the ISO and HSO samples was determined by gas chromatography (GC) in a Varian model CP3800 GC system (Varian Inc., Walnut Creek, CA) with a previous derivation of the samples to methyl ester compounds (i.e., FAME). The FFA methylation procedure consisted of adding 3 mL of 0.5% HCl in methanol to 10 mg of FFA mixture (ISO or HSO) and then heating at 70°C for 2 min [42]. The fatty acid methyl esters (FAME) of both samples were extracted with 1 mL of hexane (HPLC grade). One microliter of FAME solution in hexane was injected into a 60 m x 0.25 mm i.d., 0.25-μm film Stabilwax capillary GC column (Restek Corp, Bellefonte, PA, USA) using nitrogen as the carrier gas at a flow rate of 2 mL/min. The initial oven temperature was 150°C; subsequently, it was increased to 200°C at a rate of 10°C/min, held for 1 min, then raised to 250°C at 3°C/min and held for 20 min. The injection was in split injection mode at 250°C. A flame ionization detector (FID) (at 300°C) was used to determine the separated compounds. Individual FAs were identified by comparing their retention times to those of a FAME mixture standard (Supelco 37 Component FAME Mix, Cat. No. 47885-U, Sigma-Aldrich Quimica S.A. de C.V., Toluca, Estado de Mexico, Mexico).

#### Animals and preparation of rat aortic rings

Male Wistar rats (weight 250–350 g) were sacrificed by an overdose of pentobarbital sodium (50 mg/kg, intraperitoneal), and endothelial integrity was preserved. Upon sacrifice, the aorta was excised, cleansed of adhering tissue, and segmented into individual rings 3–4 mm wide with endothelium, according to previously established methods [43]. The Animal Care and Use Committee of the Universidad Autonoma de San Luis Potosi (Mexico) approved the use of animals and protocols established for this study (CEID2014032). The rats were maintained and sacrificed in accordance with the National Institutes of Health (NIH) Guide for the Care and Use of Laboratory Animals (Publication no. 90-23S, revised in 1985), and all efforts were made to minimize suffering.

#### Vascular response of isomerized (ISO) and hydrolyzed (HSO) samples

Individual rings with endothelium were suspended in a GRASS FT03 isometric transducer (Astro-Med, Inc., West Warwick, RI) in baths containing 5 mL of physiologic solution at 37°C. The composition (in mM) of the physiologic solution was NaCl 135, KCl 4.7, MgSO_4_ 1.17, K_2_HPO_4_ 1.152, CaCl_2_ 1.1, glucose 11.1, HEPES 18.3 and indomethacin 3 μM. The pH was set at 7.4, and as an alternative, the physiologic solution was free of gasification (O_2_/CO_2_) or bubbling [44], [45] to prevent oxidation of PUFAs (conjugated and nonconjugated).

The aortic segments were equilibrated for 1 h under a standard load of 2 g before data collection. At the end of the equilibration period, the record was readjusted to a baseline tension (i.e., 0 g of tension), and a resting tension of 2 g was maintained throughout the experimental period [46]. To evaluate the vascular response of conjugated and nonconjugated PUFAs (i.e., ISO and HSO), the vessels were precontracted with 2 μM Phe. In addition, when the contraction reached a plateau phase, the effects of ISO and HSO on the tension force in grams (g) were examined by adding them to the bath using 0.2% (v/v) DMSO as the vehicle (i.e., emulsifier), which does not affect sustained contraction by Phe [47]. The data were amplified in a GRASS P122 (Grass Instrument Co) amplifier and collected in real time using Polyview software Version 2.5 (Astro-Med, Inc., West Warwick, RI). Phe and DMSO controls were conducted to evaluate vascular reactivity. Quantitative evaluation of the vasodilator effect exerted by conjugated and nonconjugated PUFAs (i.e., ISO and HSO, respectively) on precontracted rings was measured as the percentage of reduction in the tone induced by the PUFA sample with respect to the contraction triggered by Phe. The difference (i.e., Δ Tension) between the maximum tension force induced by Phe and the relaxing force induced by the tested sample was divided by the maximum tension force (with Phe) and multiplied by 100, resulting in % relaxation.

Mixtures with different concentrations of FAs (ISO or HSO) as conjugated (mainly CEPA and CDHA) and nonconjugated FFAs (mainly EPA and DHA) were tested. Specific amounts of FFA mixtures, ISO or HSO, were diluted in physiologic solution to obtain three concentrations of EPA and DHA (in mM) in the testing bath, as follows: 1.8 mM EPA + 2.7 mM DHA (concentration A), 3.6 mM EPA + 5.4 mM DHA (concentration B), and 7.3 mM EPA + 10.8 mM DHA (concentration C). Note that when HSO was diluted, EPA and DHA were nonconjugated, but when ISO was diluted, EPA and DHA were in conjugated form (i.e., CEPA and CDHA, respectively).

#### NO production in the vascular actions induced by PUFAs

The association between the vasoactive effect and NO production promoted by conjugated or nonconjugated PUFAs (HSO or ISO) at a concentration of 1.8 mM EPA + 2.7 mM DHA upon the vascular endothelium was evaluated through the indirect determination of NO production by the quantification of nitrites following the Griess method [48]. For this purpose, 500 μL of the physiologic solution was recovered from the bath prior to contraction with Phe and after each treatment. Briefly, the samples were reduced to nitrite by bacterial nitrate-reductase enzyme (*Aspergillus niger*) donated by Instituto Potosino de Investigacion Cientifica y Tecnologica, followed by the addition of 20 μL of Griess reagent; the samples were subsequently incubated for 30 min at 37°C. Absorbance was determined at 490 nm in a Bio-Rad iMark microplate absorbance reader (Bio-Rad, Japan). The concentration of nitrites was calculated using a standard curve. In additional tests, the rings were exposed to L-NAME (0.1 mM), an inhibitor of NO production, prior to stimulation with ISO or HSO at 1.8 mM EPA + 2.7 mM DHA, and then the nitrite concentration was determined as previously described.

#### The role of endothelium in the vascular actions induced by ISO

In order to evaluate the role of endothelium in the vascular dilation induced by ISO, the endothelium was kept (+E) or removed (-E) by gently rubbing the vessel lumen with a cotton swab; after this procedure, the physiological protocols were applied as previously described. ACh was used as a positive control of relaxation, whose mechanism of action depends on both the endothelium and endothelial NO production. The effectiveness of removal of the endothelium was confirmed through the absence of relaxation or the maintenance of the contractile effect [49]. Sustained relaxation reported as more than 70% of precontracted tone in the presence of ACh ensures the presence of endothelium [50].

#### Statistical analysis

The effects of conjugation of the double bonds of n-3 PUFAs and of the concentration of FAs on vascular response (measured as % relaxation) were evaluated by a two-factor analysis of variance (ANOVA). The factors were the conjugation of PUFAs (two levels: conjugated and nonconjugated PUFAs, ISO and HSO, respectively) and the concentration of fatty acids (three levels: A, 1.8 mM EPA + 2.7 mM DHA; B, 3.6 mM EPA + 5.4 mM DHA; and C, 7.3 mM EPA + 10.8 mM DHA). Triplicates were performed for each treatment. Two-way ANOVA was followed by a *post hoc* Tukey test to detect significant differences at P< 0.05 among treatments. Statistical analysis was performed using GraphPad Prism V 5.01 for Windows (GraphPad Software, San Diego California, USA).

## Results and discussion

The present work shows the effect of a mixture of conjugated or nonconjugated isomers of n-3 PUFAs, mainly DHA and EPA, on isolated rat aortic rings. Both ISO and HSO exhibited vasodilation activity; however, NO production as a signaling molecule that induced this effect is not shared by ISO, suggesting different mechanisms of action to promote relaxation. The only difference between both HSO and ISO samples is the presence of double bonds in conjugated systems in the PUFAs of ISO.

### Fatty acid composition

UV-Vis spectrophotometric analysis confirmed the formation of conjugated isomers (i.e., conjugated dienes, trienes, tetraenes and pentaenes) in ISO during the alkaline isomerization reaction. The composition of conjugated isomers in ISO and HSO samples is shown in Table 1. There was a small amount (2%) of conjugated isomers in the hydrolyzed sample (HSO).

**Table 1:** Conjugated isomer composition (mean ± standard deviation) of hydrolyzed shark oil (HSO) and isomerized shark oil (ISO).

	Conjugated isomers % (w/w)				
	Dienes	Trienes	Tetraenes	Pentaenes	Total conjugated
**HSO**	1.17 ± 0.13	0.21 ± 0.11	0.43 ± 0.12	0.19 ± 0.02	2.0 ± 0.36
**ISO**	23.32 ± 0.71	3.89 ± 1.11	6.03 ± 0.15	4.09 ± 0.88	37.33 ± 1.14

The result of the isomerization process of shark oil was that the total amount of conjugated isomers increased from 2.0 in HSO to 37.3% in ISO. The conjugated fraction (i.e., 37.3%) consisted of 62.5% conjugated dienes, 10.4% conjugated trienes, 16.1% conjugated tetraenes and 10.9% conjugated pentaenes.

A previous study on the anticarcinogenic effect of conjugated EPA (CEPA) reported that alkaline isomerization of EPA (99% purity) produced 57.6% conjugated dienes, 34.5% conjugated trienes, 6.7% conjugated tetraenes and 1.2% conjugated pentaenes [51]. In the present work, a higher formation of conjugated dienes and a lower content of conjugated trienes were observed. However, it should be considered that the isomerized sample in this study was a mixture of all the FAs present in shark oil, which includes several PUFAs, mainly EPA and DHA, which are prone to undergo conjugation of double bonds.

The FA composition of samples of HSO and ISO determined by GC is given in Table 2.

**Table 2:** FA composition (mean ± standard deviation) of hydrolyzed shark oil, HSO (before alkali-catalyzed isomerization), and isomerized shark oil (ISO).

Fatty Acid	(%)	
	HSO	ISO
Myristic (C14:0)	4.65 ± 0.18	4.52 ± 0.15
Palmitic (C16:0)	18.81± 1.07	19.14 ± 1.29
Palmitoleic (C16:1)	9.08 ± 0.26	8.79 ± 0.32
Stearic (C18:0)	4.39 ± 0.13	4.47 ± 0.31
Oleic (C18:1)	14.8 ± 0.23	15.04 ± 0.42
Linoleic (C18:2), n-6	1.05 ± 0.30	0.98 ± 0.10
Linolenic (C18:3), n-6	1.62 ± 0.12	0.25 ± 0.17
Eicosenoic (C20:1)	4.91 ± 0.64	4.80 ± 0.24
Arachidonic (C20:4), n-6	3.68 ± 0.09	0.36 ± 0.15
Eicosapentaenoic (C20:5), n-3	10.30 ±0.75	0.32 ± 0.15
Lignoceric (C24:0)	2.01 ± 0.08	2.12 ± 0.21
Docosapentaenoic (C22:5), n-6	4.24 ± 0.14	0.26 ± 0.16
Docosahexaenoic (C22:6), n-3	18.60 ± 1.48	0.34 ± 0.17
Saturated	29.86 ± 1.34	30.25 ± 1.03
Monounsaturated	28.79 ± 1.52	28.63 ± 2.58
Polyunsaturated (nonconjugated PUFAs)	39.49 ± 1.98	2.51± 0.98

The effect of the isomerization process on the FA content of shark oil was the conversion or isomerization of all PUFAs present in the oil (i.e., linoleic, linolenic, arachidonic, eicosapentaenoic, docosapentaenoic and docosahexaenoic acids) to conjugated isomers. This can be seen as the corresponding decrease in the content of nonconjugated PUFAs in ISO. Only PUFAs can undergo such isomerization because the number of double bonds necessary to form a conjugated system is more than one. It is important to note that DHA and EPA constitute 73% of the total PUFAs and that other PUFAs, such as linoleic, linolenic, arachidonic, and docosapentaenoic acids, are n-6 fatty acids. This can exert some effect on the measurements in this study.

The decrease observed in the PUFA content of ISO was due to the formation of conjugated isomers, as shown in Table 1. However, the content of all other FAs (i.e., saturated and monounsaturated FAs, Table 2) remained constant after the alkaline treatment, indicating that the only change during the isomerization process was the conjugation of double bonds in PUFAs.

According to Table 1, a total amount of 37.33% of conjugated isomers was formed through the isomerization process, which means that approximately 94.5% of the total PUFAs in shark oil (39.49% of FAs in HSO, Table 2) was isomerized to conjugated molecules, while approximately 2.5% of PUFAs remained not isomerized.

**Fig 1:**
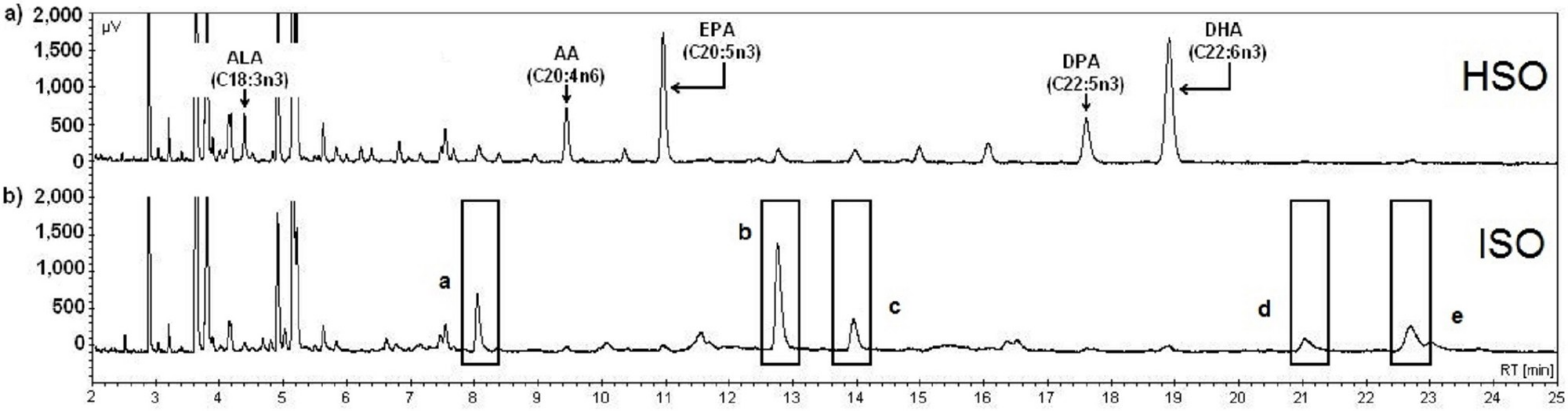
Fatty acid chromatograms of the shark oil samples. a) Hydrolyzed shark oil, HSO (shark oil before alkali-catalyzed isomerization), and b) isomerized shark oil, ISO, resulting from alkali-catalyzed isomerization.

Additionally, representative chromatograms of HSO and ISO are shown in Fig 1 as a complement to Tables 1 and 2. For HSO (Fig 1a), the peaks corresponding to the nonconjugated PUFAs [i.e., the fatty acids linolenic (ALA), arachidonic (AA), eicosapentaenoic (EPA), docosapentaenoic (DPA), and docosahexaenoic (DHA)] are indicated by arrows. The peaks of these FAs considerably decreased after the alkaline treatment (Fig 1b) due to their conversion to conjugated isomers. Furthermore, in the chromatogram for ISO (Fig 1b), some increased or new peaks that primarily correspond to the compounds produced during isomerization (i.e., conjugated isomers) are marked (with boxes, a-e). GC could not identify such compounds with the standard used since the standard mixture consisted only of nonconjugated FAs.

On the other hand, the relatively high concentration of n-3 PUFAs, mainly EPA (10.3%) and DHA (18.6%) found in the oil, makes the liver of *Carcharhinus falciformis* an interesting source of oil. As with other marine sources, shark oils are diverse and complex chemical structures ranging from saturated to polyunsaturated fatty acids. The number of carbons in the fatty acid chain is 20, 22 or even 24. EPA and DHA account for 15% to 30% of the marine oils. In this respect, the proportion varies from fish to fish since the food sources change according to geographical regions. PUFAs are synthetized by phytoplankton and pass up the food chain through zooplankton to small and medium fishes and crustaceans, which are prey of sharks [52]. Silky shark species such as *Carcharhinus falciformis* contain high levels of fatty acids, which accumulate during periods of essential fatty acid deficiency. Despite the lack of information on the diet and overall characterization of PUFAs in the shark liver, the squid *Dosidicus gigas* and other crustaceans and fishes could contribute to the high n-3 and n-6 contents in *C*. *falciformis* [53].

### Vascular response of HSO and ISO

The effect of EPA and DHA on the endothelium has been widely described in the literature [24], [30], [54], [55]. Considering that conjugation of double bonds in n-3 PUFAs (CEPA and CDHA) increases the anticarcinogenic effect of EPA and DHA [33]–[38], [56], it is expected that conjugation of double bonds of n-3 PUFAs exhibits a similar or higher effect on the vascular tone than nonconjugated n-3 PUFAs. Thus, mixtures of FAs, HSO and ISO, containing high concentrations of n-3 EPA and DHA or CEPA and CDHA, respectively, were evaluated based on the vascular responses of precontracted rat aortic rings.

**Fig 2:**
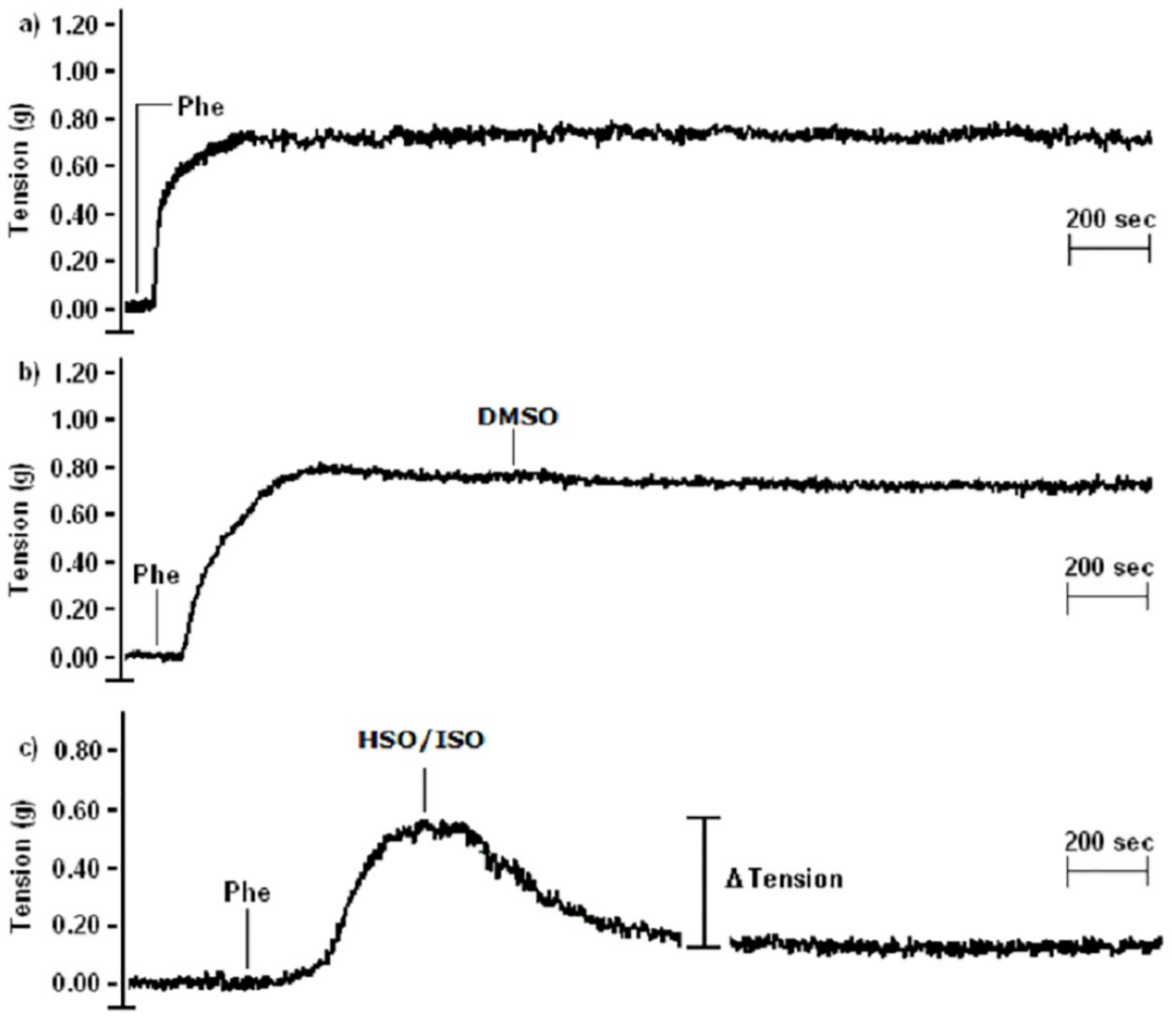
The effect on the tension force of rat aortic rings induced by vasoactive substances. a) Phenylephrine (Phe) promoted vasoconstriction, b) DMSO did not exert an effect, and c) either HSO (hydrolyzed shark oil) or ISO (isomerized shark oil) induced vasorelaxation. Tension, in grams (g), was considered an index of the vascular tone. Recordings are representative of three independent experiments.

First, Fig 2 shows representative recordings of different tests conducted to demonstrate 2a) that the contraction induced by Phe was maintained in the control experiment; 2b) that DMSO (used as a vehicle for FFAs) does not affect the contraction induced by Phe; and 2c) the typical behavior (i.e., relaxation effect) exerted by either ISO or HSO. This indicates that n-3 PUFAs (in HSO or ISO) *per se* promote vasodilation. Such a relaxation effect was induced with different intensities, depending on the treatment, which was defined by the conjugation or nonconjugation, and by the level of DHA and EPA concentration (i.e., concentration levels A, B or C of DHA and EPA).

The relaxation effect was measured as the percentage of reduction of the tone induced by Phe (2 μM) exerted by the tested sample (ISO or HSO). Thus, the difference (i.e., Δ Tension, Fig 2c) between the maximum tension force induced by Phe and the relaxing force induced by the tested sample was divided by the maximum tension force induced by Phe and multiplied by 100, resulting in % relaxation.

The fact that mixtures with nonconjugated HSO rich in EPA and DHA exhibited a vasorelaxation response on aortic rings was attributable to the presence of such FAs. These results agree with previous reports, which concluded that EPA induces relaxation and antagonizes the contraction of precontracted bovine coronary arteries [23], [57], while other findings in animals [27], [30] and humans [28], [29] indicated that DHA plays an important role in endothelial function, which beneficially affects cardiovascular health.

Regarding the use, in this study, of a physiologic solution free of gasification (O_2_/CO_2_) or bubbling, which was considered as an alternative to prevent oxidation of PUFAs, in contrast with the majority of other reported studies [25], [49], it seems not to affect the results because of the similarity in physiologic profile observed for nonconjugated PUFAs (i.e., HSO). The use of tissues under gasification is common because under limited time, it maintains the viability of the tissue [27]. Physiological systems need to have precise control of the bubbling system; in other words, they need to have a constant flow and be homogenous to maintain stability [44]. However, oxygenation does not seem to be critical for most isolated blood vessel preparations unless they become anoxic for a considerable period of time. It may be of more importance in the study of pulmonary or umbilical cord vessels, which are most sensitive to alterations in the pO_2_ of the surrounding medium [45].

On the other hand, the vasorelaxation effect of a mixture of FAs containing high concentrations of conjugated PUFAs, mainly CEPA and CDHA, among other FAs, on vascular tone is reported for the first time. Because the primary components of such a mixture are CEPA and CDHA (approx. 30% of the total FAs), the vasorelaxation effect shown by ISO is mainly attributable to such FAs. However, the effect of the other FAs present in the mixture, mainly PUFAs (i.e., n-6), should not be discarded.

**Fig 3:**
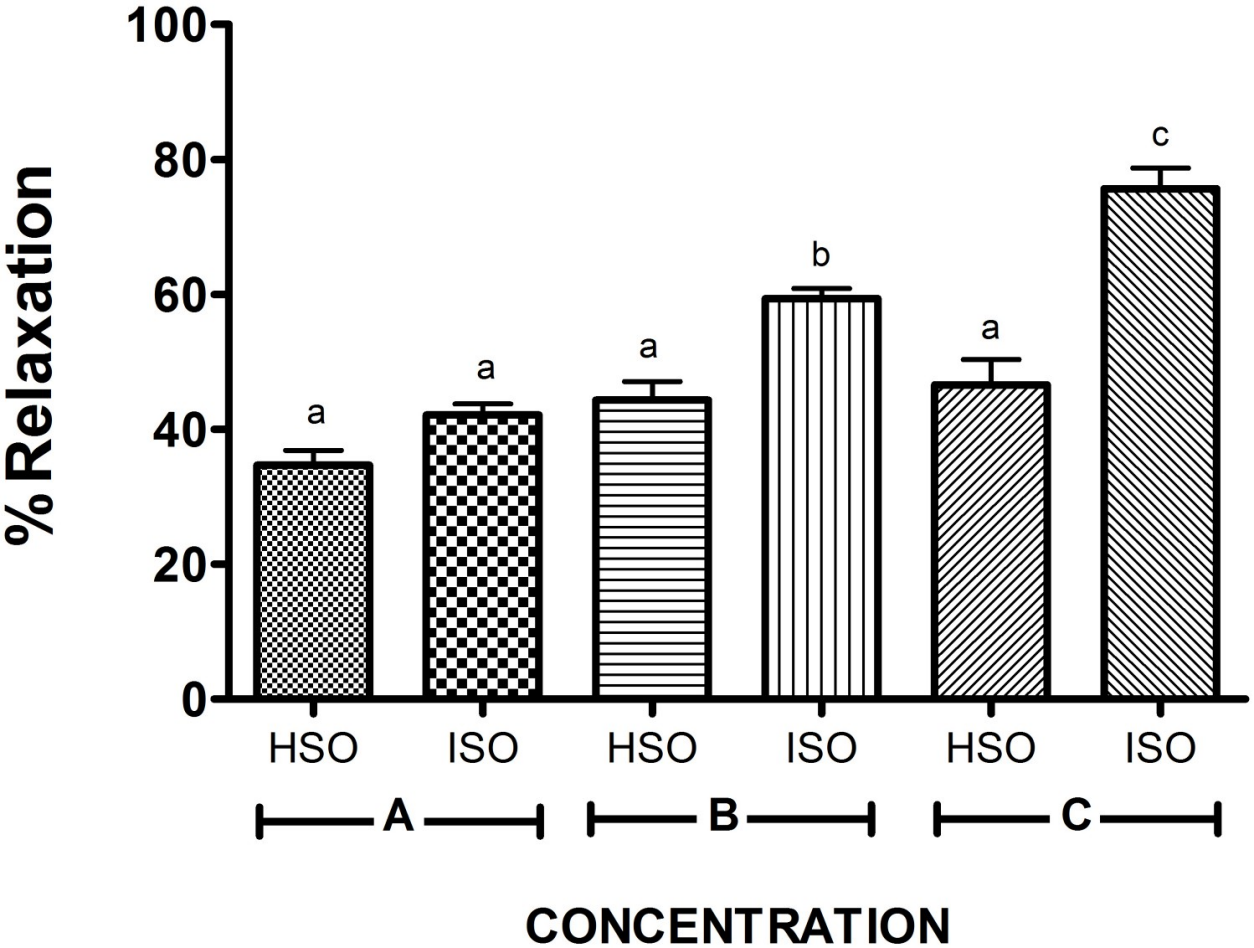
The effect on vasorelaxation activity (% relaxation) induced by isomerized (ISO) and hydrolyzed samples (HSO) at different concentrations of PUFA (in mM). Concentration A: 1.8 EPA + 2.7 DHA, concentration B: 3.6 EPA + 5.4 DHA, and concentration C: 7.3 EPA + 10.8 DHA. Values are shown as the means ± SD, n = 3. Means without a common letter differ (P < 0.05).

As expected, such an effect (i.e., % relaxation) with ISO was higher than that of the nonconjugated isomers (HSO). ANOVA tests detected significant effects of the conjugation of PUFAs (P<0.001) and their concentration (P<0.001) on vasorelaxation activity. These results are presented in Fig 3. All the treatments (HSO or ISO) showed a vasodilator effect (% relaxation > 0). Compared to HSO, ISO displayed a significantly increased relaxation effect, which increased with concentration. No significant differences were found at different concentrations of HSO.

These results demonstrate that conjugation of PUFAs present in ISO, mainly CEPA and CDHA, increases the vasorelaxation effect of such nonconjugated FAs in a dose-dependent manner, as the only difference between ISO and HSO is that PUFAs in the first are conjugated, while in the second, PUFAs are nonconjugated. This improvement in relaxation activity provides a new physiological profile of such conjugated isomers (CEPA and CDHA) since they show higher effects than their nonconjugated counterparts. Thus, they could improve the physiological effects in CVD or could be used in minor doses to exert similar relaxant activity.

Regarding the concentrations of PUFAs used in this work and their relation to pharmacokinetic studies reported by other authors, Braeckman et al. [58] studied EPA pharmacokinetics by oral dosing in healthy subjects. Braeckman’s group and other authors [58]–[60] have observed that EPA is not detectable in blood following oral intake; however, it is detectable when EPA has been hydrolyzed during digestion as free fatty acid. Braeckman et al. reported that “the mean plasma total EPA increased from 19 μg/mL to a peak (C_max_) of 366 μg/mL at 5 hours postdosing 4 g/day of IPE (icosapent ethyl (IPE) is a prescription form of eicosapentaenoic acid (EPA) ethyl ester) on day 28. The mean RBC EPA Cmax after 4 g/day was 89 μg/mL (baseline, 12 mg/mL). Means in the steady state (SD) for half-life, clearance, and volume of distribution of total EPA were 79 (47) hours, 757 (283) mL/h, and 82 (56) L, respectively. Steady state for total and unesterified plasma EPA was reached by day 28, whereas RBC levels were still increasing.” The EPA pharmacokinetic profile demonstrated a slowly cleared, extensively distributed molecule with dose linearity and comparable exposures with BID and QD regimens, and in regard to daily exposure, comparable results for once- and twice-daily IPE regimens and 500 and 1000 mg formulations at comparable doses were observed with no effects of age and minimal effects of sex. The lowest concentration of EPA used here was 1.8 mM (or 544.41 μg/mL). The lowest concentration of DHA was 2.7 mM (or 886.917 μg/mL), which is in the range of C_max_ observed for EPA reported previously (366 μg/mL) [58].

In addition, Libianki et al. [61] showed the bioavailability of various formulations of n-3 PUFAs and tocopheryl phosphate mixture (TPM) following oral administration in rats and assessed whether TPM could improve the oral absorption of DHA, reporting that the ability of TPM to increase DHA bioavailability was replicated at higher doses of DHA. The Tmax values for DHA doses (high and low) remained similar; however, differences in C_max_ were observed in groups with high DHA doses; specifically, when TPM was administered, the C_max_ value was approximately double that of the group with the high DHA dose alone (C_max_ of 124.8 μg/mL). The group evaluated with a high DHA dose resulted in a mean C_max_ of 160.5 μg/mL when TPM was administered in a 1:0.1 ratio, and the plasma DHA profile was approximately 30% higher than that of the high DHA dose control group (no TPM), indicating that TPM could enhance the bioavailability of DHA [61].

Additionally, we used higher concentrations of EPA and DHA, which despite being considered supraphysiological, could provide relevant information about the potential mechanisms of action associated or not with the production of NO.

Unlike those studies that investigated the relaxation effect of individual high-purity PUFAs (i.e., nonconjugated EPA or DHA), and the anticarcinogenic effect of nonconjugated and conjugated PUFAs (EPA or DHA), the systems investigated here, besides EPA and DHA, consisted of mixtures of several FAs naturally present in the shark oil, including saturated, monounsaturated and n-6 polyunsaturated fatty acids (Table 2). Therefore, the vasodilator effect of PUFAs observed in both HSO and ISO in this study can be considered as the sum of the individual effects, either vasodilator or vasoconstrictor, of each FA present in each mixture, where vasorelaxation was the predominant effect, possibly due to the predominant presence of both EPA and DHA.

Additionally, we compared the % relaxation induced by the systems of this study (i.e., ISO and HSO at concentrations A, B, and C of EPA and DHA isomers) with samples of ISO and HSO at lower levels, on the order of 100 μM of each n-3 PUFA (i.e., EPA and DHA), and with the % relaxation of pure standards of EPA and DHA in the conjugated and nonconjugated forms, alone and combined (EPA and DHA), at the lowest level (A concentration) and in the range of 100 μM. Such data resulted from preliminary experiments of another work (S1 Text).

First, we observed that conjugated-PUFAs (HSO) (Fig 1B and Fig 1C in S1 Text) induced a higher relaxation than the nonconjugated form (Fig 1A and Fig 1C in S1 Text) in the presence of endothelium (+E). This indicates similar results to those observed at higher levels of PUFAs (i.e., concentrations A, B, and C).

Additionally, data resulting from preliminary experiments of such work for the % relaxation of samples of pure standards of EPA and DHA in the conjugated and nonconjugated forms, alone and combined (EPA and DHA), were analyzed. When comparing such preliminary results against the % relaxation induced by the systems of this study (i.e., ISO and HSO at concentrations A, B, and C of DHA and EPA isomers) (Fig 3 in S1 Text), it can be summarized, in the context of this study, as follows. 1) All pure standard samples exerted a relaxation effect, which can be as intense as that of ISO and HSO mixtures. 2) Conjugation of individual standards does not seem to have the same impact on the relaxation activity of pure samples of DHA and EPA as samples of HSO and ISO. 3) EPA alone seems to induce higher relaxation than DHA. 4) When DHA and EPA are combined, they seem to exert a synergistic effect. Except for what is mentioned in point one, the other points generate an interesting surprise. It allows us to infer that each FA (EPA or DHA) shows a different effect and that other FAs present in HSO and ISO induce a critical impact on vascular tone. Interestingly, the global impact of the mixtures (i.e., HSO and ISO) was relaxation, which is favorable. This evidence indicates that the influence of EPA and DHA was predominant over the impact of the other FAs. However, the effect of the other FAs should not be ignored and should also be studied.

Examination of the vascular effect of an isomerized sample of fish oil is attractive from the industrial point of view because, although the methods of isolation and purification of EPA and DHA are being developed to provide highly purified PUFAs for research in human health [27]–[29] and animals [23], [30], [57], the cost of separation and concentration is high. It is likely that commercial access to EPA or DHA and their corresponding conjugated isomers will remain as a mixture rather than purified compounds. If pursued for a practical purpose, the investigation of the isomerization process and/or physiological effects of conjugated FAs should be focused on the effect of conjugated isomers with FAs obtained from the isomerization reaction of an oil rich in n-3 PUFAs instead of pure samples.

### Role of NO in the effects of HSO and ISO

To date, it is known that the mechanisms by which EPA induces endothelium-dependent and endothelium-independent vasorelaxation are *via* NO production, prostaglandins and calcium-activated potassium channels [23], [30], [54], [57], [62]. However, the mechanisms by which CPUFAs induce relaxation of blood vessels are still unknown. Thus, establishing the mechanism of action by which conjugated PUFAs produce such effects and the role of the endothelium will allow the development of new pharmacological tools.

**Fig 4:**
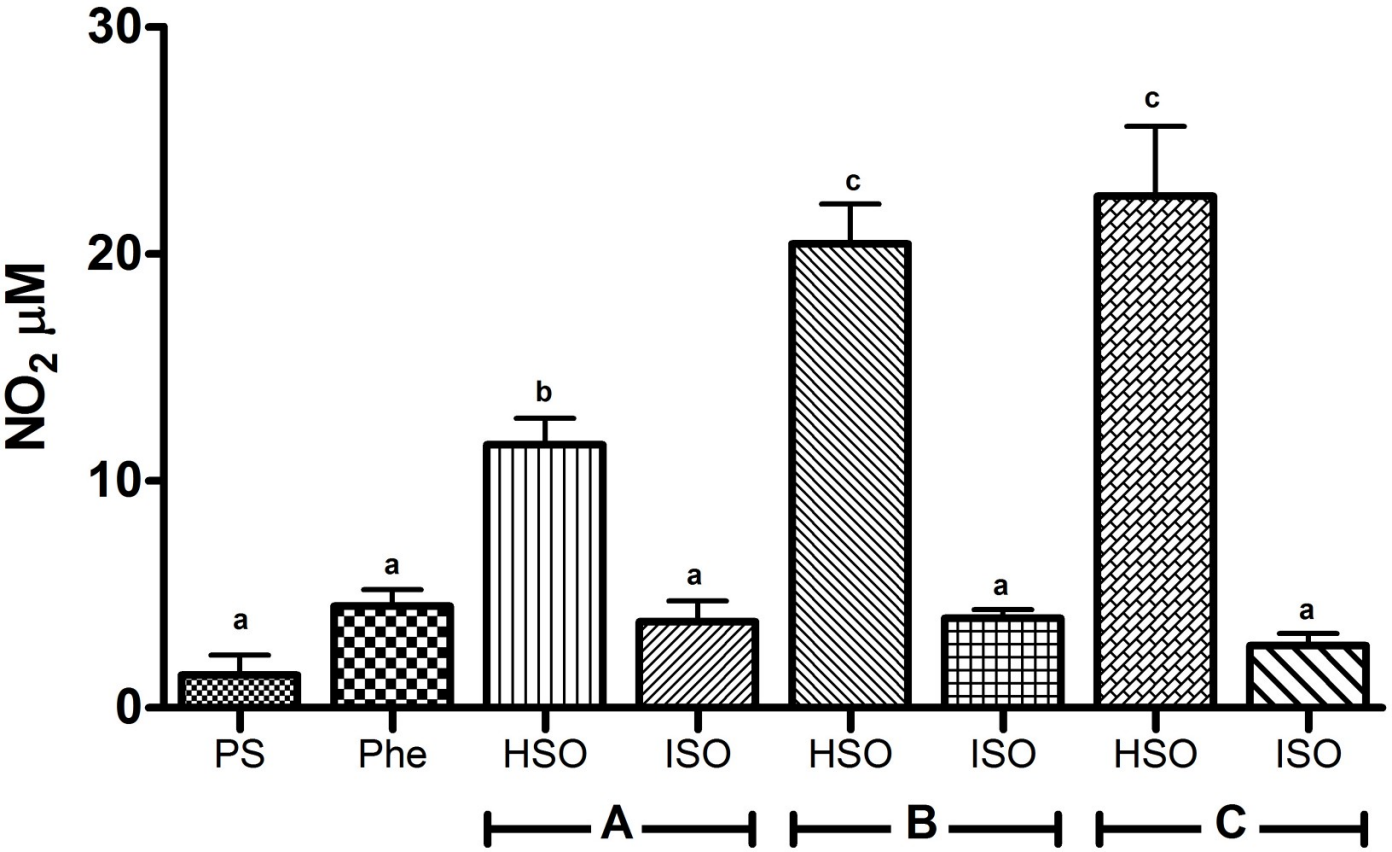
Production of NO by isomerized shark oil (ISO) and hydrolyzed shark oil (HSO) at different concentrations (in mM). Concentration A: 1.8 EPA + 2.7 DHA, concentration B: 3.6 EPA + 5.4 DHA, and concentration C: 7.3 EPA + 10.8 DHA. In addition, the treatments of HSO and ISO are compared to physiologic solution (PS) and phenylephrine (Phe) used as controls of basal NO production. Values are shown as the means ± SD, n = 3. Means without a common letter differ (P<0.05).

In this study, the concentration of nitrites as an indicator of the involvement of NO as one of the mediators in the relaxing vascular effects was determined. Nevertheless, the results (Fig 4) show that hydrolyzed samples (HSO) produced NO in a dose-dependent manner, while isomerized samples (ISO) did not modify the production of this metabolite. Thus, vasorelaxation induced by ISO is not dependent on NO production, suggesting that other signaling molecule(s) is/are involved in the regulation of vascular tone.

**Fig 5:**
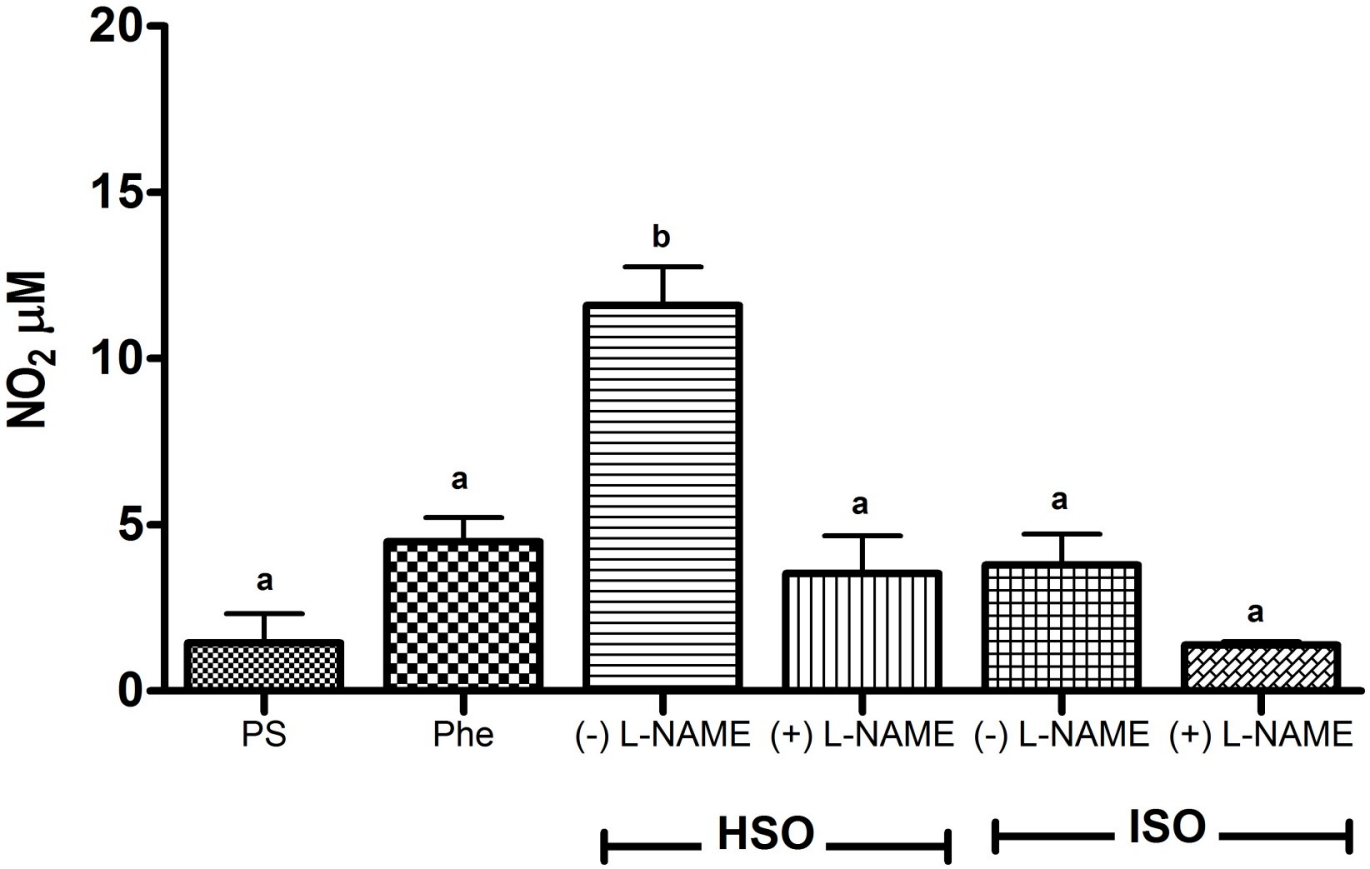
Effect of L-NAME on the production of NO by isomerized shark oil (ISO) and hydrolyzed shark oil (HSO). Concentration A: 1.8 EPA + 2.7 DHA with and without L-NAME. In addition, the treatments of HSO and ISO are compared to PS and Phe, used as controls of basal NO production. Values are shown as the means ± SD, n = 3. Means without a common letter differ (P < 0.05).

**Fig 6:**
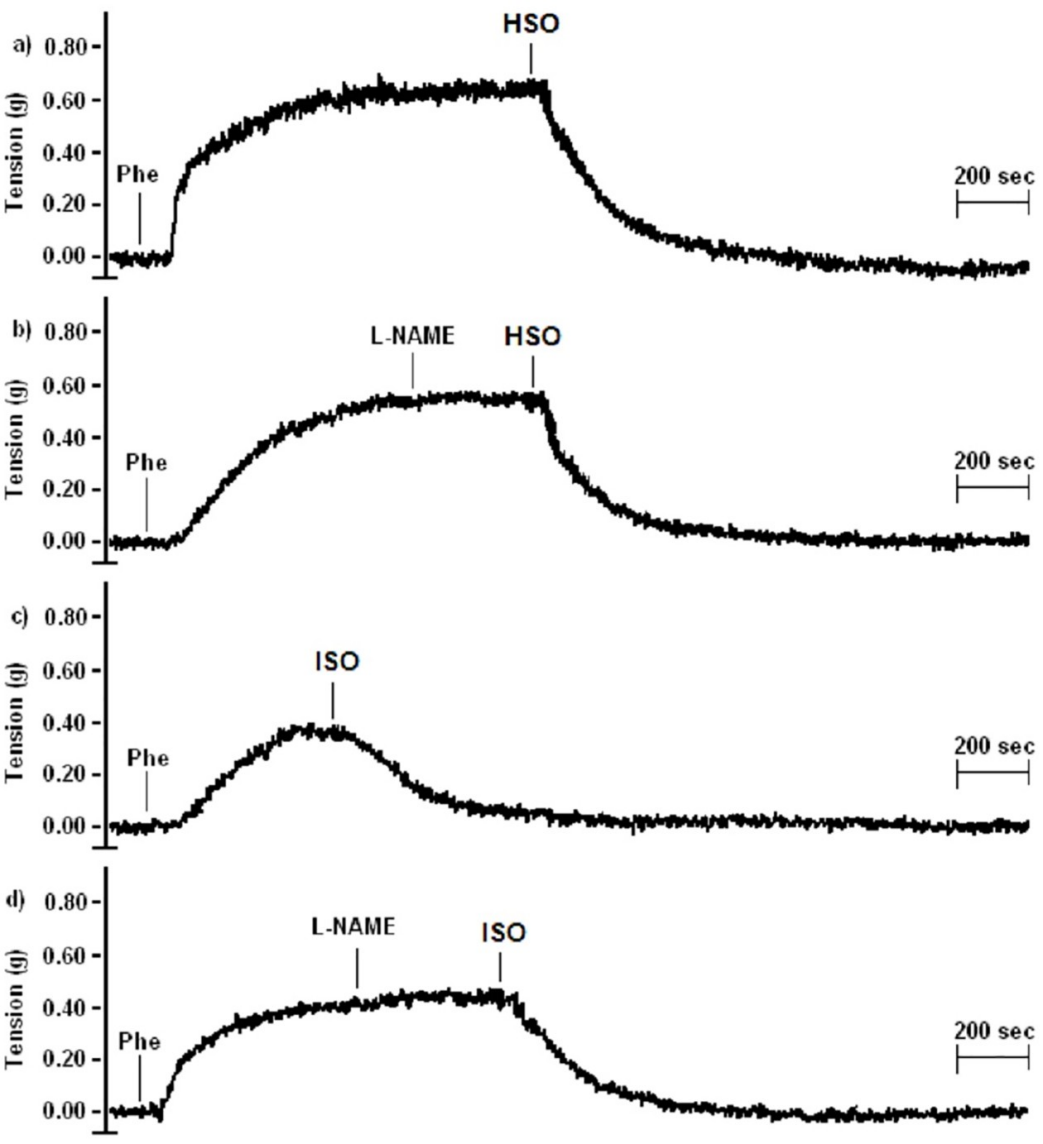
Effect of hydrolyzed (HSO) and isomerized shark oil (ISO) on vascular tone, with or without L-NAME. Vasorelaxation induced by HSO with (a) and without (b) L-NAME and of ISO with (c) and without (d) L-NAME at concentration A: 1.8 EPA + 2.7 DHA. The tension in grams (g) was considered an index of the vascular tone.

In the treatments with or without L-NAME, an inhibitor of NO production, the expected NO suppression was observed when HSO was added, as HSO induced NO production (Fig 5). Such an effect was not observed when ISO was added because ISO does not induce NO production. However, the vasoactive response of hydrolyzed and isomerized samples at concentration A (1.8 EPA mM + 2.7 DHA mM) was not inhibited (Fig 6).

### Effect of the endothelium on the vasodilation induced by ISO

**Fig 7:**
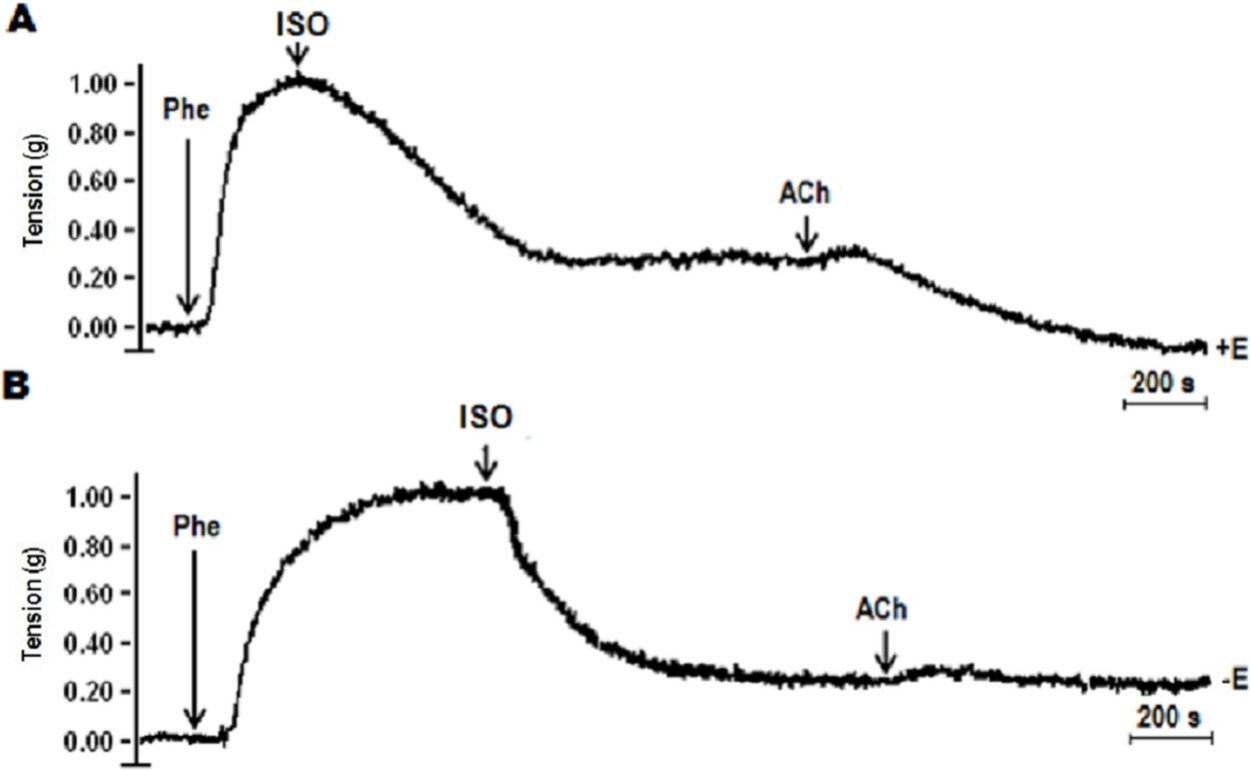
Effect of endothelium removal on the tension force of rat aortic rings induced by ISO. a) ISO induced vasorelaxation in the presence of endothelium (+E), b) ISO induced vasorelaxation in the absence of endothelium (-E). The tension in grams (g) was considered an index of the vascular tone. ACh was taken as a control of vasorelaxation dependent on endothelium (+E). Recordings are representative of three independent experiments at a concentration of 1.8 mM EPA + 2.7 mM DHA.

In order to evaluate whether the endothelium could be a key factor involved in the relaxation effects induced by ISO, the endothelium was removed, and vasodilation was compared to treatments with endothelium (Fig 7). ISO induced vasorelaxation at similar magnitudes in both conditions, suggesting that both the endothelium (Fig 7) and consequently NO production are not involved in the reaction mechanism of the relaxant effect induced by ISO (Fig 5) and that other different signaling molecule(s) at the smooth muscle level could regulate vascular tone by ISO. Moreover, hydrolyzed samples produced dose-dependent NO since it was inhibited by L-NAME, but the vasorelaxation induced by hydrolyzed samples was not blocked. It is possible that the relaxant effect of the hydrolyzed sample has been induced by two mechanisms, one in which the production of NO is involved in a lesser manner and another, NO-independent, which may be exerting the greatest dilator effect.

Previously, an *in vivo* study showed that the endothelium-dependent vascular response exerted by acetylcholine (ACh) in healthy men and women was significantly improved after tuna oil supplementation, a fatty acid mixture consisting of 6% EPA and 27% DHA [63]. Therefore, an endothelium-dependent vasodilator response of CEPA and CDHA during their supplementation could be expected. Hence, the use of CEPA and CDHA in functional foods and treatments is likely to have a great future after its safety is confirmed in long-term safety studies and clinical trials.

Similarly, Limbu et al. [30] performed a study with both nonconjugated PUFA EPA and DHA in aorta and mesenteric artery rings. The nonconjugated fatty acids induced partial vasodilation in mesenteric arteries with no endothelium. The authors concluded that CYP450 (cytochrome P450) and IKCa (K+ and Na+ channel) receptors are related to the induction of vasodilation by EPA in the mesenteric artery, while IKCa could be implicated in the relaxation exerted by DHA in the aorta [30]. Takenouchi et al. [64] studied the effect of EPA ethyl ester (EPA-E) supplementation in the diabetic KKAy (transcription factor kayak) mouse and nondiabetic C57BL/6 mouse (10 mg/day, 4 weeks). Interestingly, after treatment, aorta rings isolated from nondiabetic C57BL/6 mice fed EPA-E improved the vasodilation exerted by ACh. Additionally, the enhanced vasocontractile effect of Phe in aorta rings isolated from diabetic KKAy mice was not modified by the administration of EPA-E. Direct administration of EPA and other metabolites induced relaxation in the aorta ring isolated from C57BL/6J mice. The relaxation activity is reported by the authors as endothelium independent [64].

## Conclusions

The liver of shark (*Carcharhinus falciformis*) is a suitable source of marine oil rich in n-3 PUFAs (mainly EPA and DHA). The present study has demonstrated, for the first time, that conjugation of double bonds of PUFAs (EPA and DHA) increases the vasodilator activity of such conjugated FAs on rat aortic rings. The data open new avenues for the study of these mixtures in the prevention and treatment of cardiovascular diseases. In addition, nonconjugated PUFAs produce NO in a dose-dependent manner, while conjugated PUFAs do not, suggesting that their dilator mechanism is independent or partially independent of NO. It is still necessary to study the effect of the conjugation of double bonds of pure n-3 PUFAs (DHA and EPA) to evaluate the contribution of each one in a mixture with other FAs and in less complicated systems, such as model systems, to explain the effects observed in this study.

## Supporting information

S1 Raw images (PDF)

S1 Text The % relaxation induced by ISO and HSO at lower levels, on the order of 100 μM of each n-3 PUFA (i.e., EPA and DHA), and the % relaxation of pure standards of EPA and DHA in the conjugated and nonconjugated forms, alone and combined (EPA and DHA). (DOCX)
