# Supplementary material for: Effect of the double bond conjugation on the vascular physiology and nitric oxide production of isomers of eicosapentaenoic and docosahexaenoic acids prepared from shark oil
Source: PLoS One. 2020 Feb 27;15(2):e0229435. doi: 10.1371/journal.pone.0229435 (PMC7046235; doi:10.1371/journal.pone.0229435)
Supplement: S1 Raw images — (PDF) [file pone.0229435.s001.pdf]

Vascular response of HSO and ISO
From GraphPad Prism
(Fig 3)

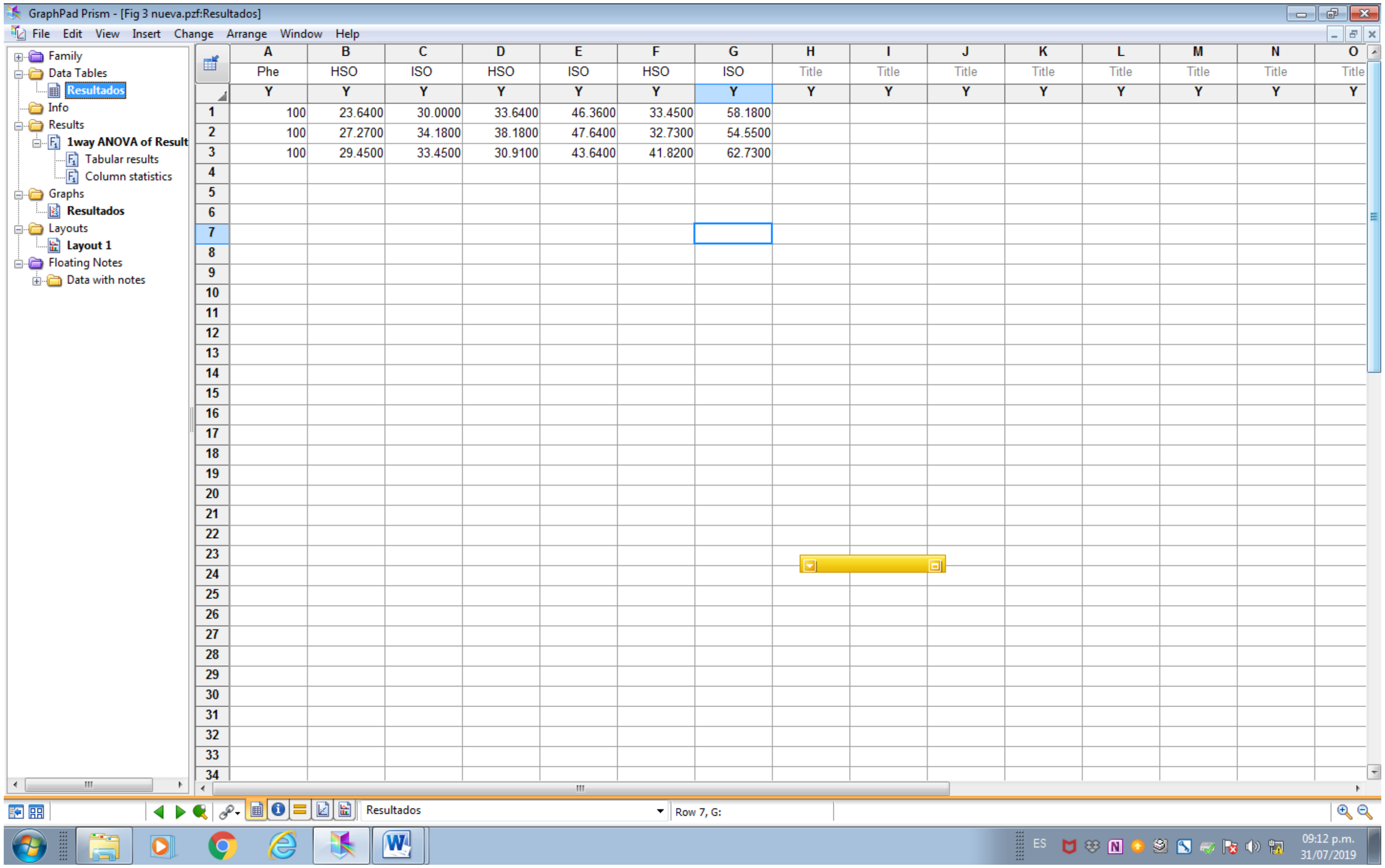

	A	B	C	D	E	F	G
	Phe	HSO	ISO	HSO	ISO	HSO	ISO
	Y	Y	Y	Y	Y	Y	Y
1	100	23.6400	30.0000	33.6400	46.3600	33.4500	58.1800
2	100	27.2700	34.1800	38.1800	47.6400	32.7300	54.5500
3	100	29.4500	33.4500	30.9100	43.6400	41.8200	62.7300

GraphPad Prism - [Fig 3 nueva.pzf:1way ANOVA of Resultados]

File Edit View Insert Change Arrange Window Help

- Family
- Data Tables
  - Resultados
- Info
- Results
  - 1way ANOVA of Result
    - Tabular results
    - Column statistics
- Graphs
  - Resultados
- Layouts
  - Layout 1
- Floating Notes
  - Data with notes

1way ANOVA Column statistics	A Phe	B HSO	C ISO	D HSO	E ISO	F HSO	G ISO	H Title	I Title	J Title	K Title	L Title	M Title
	Y	Y	Y	Y	Y	Y	Y	Y	Y	Y	Y	Y	Y
1 Number of values	3	3	3	3	3	3	3						
2													
3 Minimum	100.0	23.64	30.00	30.91	43.64	32.73	54.55						
4 25% Percentile	100.0	23.64	30.00	30.91	43.64	32.73	54.55						
5 Median	100.0	27.27	33.45	33.64	46.36	33.45	58.18						
6 75% Percentile	100.0	29.45	34.18	38.18	47.64	41.82	62.73						
7 Maximum	100.0	29.45	34.18	38.18	47.64	41.82	62.73						
8													
9 Mean	100.0	26.79	32.54	34.24	45.88	36.00	58.49						
10 Std. Deviation	0.0	2.935	2.233	3.672	2.043	5.053	4.099						
11 Std. Error	0.0	1.695	1.289	2.120	1.179	2.917	2.366						
12													
13 Lower 95% CI	100.0	19.50	27.00	25.12	40.81	23.45	48.31						
14 Upper 95% CI	100.0	34.08	38.09	43.37	50.95	48.55	68.67						

1way ANOVA of Resultados | Column statistics

ES 09:13 p.m. 31/07/2019

GraphPad Prism - [Fig 3 nueva.pzf:1way ANOVA of Resultados]

File Edit View Insert Change Arrange Window Help

- Family
- Data Tables
  - Resultados
- Info
- Results
  - 1way ANOVA of Resulta
    - Tabular results
    - Column statistics
- Graphs
  - Resultados
- Layouts
  - Layout 1
- Floating Notes
  - Data with notes

1way ANOVA
Tabular results

16	Is there significant matching? (P < 0.05)	No				
17						
18	ANOVA Table	SS	df	MS		
19	Treatment (between columns)	11520	6	1920		
20	Individual (between rows)	20.07	2	10.04		
21	Residual (random)	127.1	12	10.59		
22	Total	11670	20			
23						
24	Tukey's Multiple Comparison Test	Mean Diff.	q	Significant? P < 0.05?	Summary	95% CI of diff
25	Phe vs HSO	73.21	38.96	Yes	***	63.91 to 82.51
26	Phe vs ISO	67.46	35.90	Yes	***	58.16 to 76.76
27	Phe vs HSO	65.76	34.99	Yes	***	56.46 to 75.06
28	Phe vs ISO	54.12	28.80	Yes	***	44.82 to 63.42
29	Phe vs HSO	64.00	34.06	Yes	***	54.70 to 73.30
30	Phe vs ISO	41.51	22.09	Yes	***	32.21 to 50.81
31	HSO vs ISO	-5.757	3.064	No	ns	-15.06 to 3.545
32	HSO vs HSO	-7.457	3.968	No	ns	-16.76 to 1.845
33	HSO vs ISO	-19.09	10.16	Yes	***	-28.39 to -9.792
34	HSO vs HSO	-9.213	4.903	No	ns	-18.51 to 0.08792
35	HSO vs ISO	-31.70	16.87	Yes	***	-41.00 to -22.40
36	ISO vs HSO	-1.700	0.9047	No	ns	-11.00 to 7.601
37	ISO vs ISO	-13.34	7.098	Yes	**	-22.64 to -4.035
38	ISO vs HSO	-3.457	1.840	No	ns	-12.76 to 5.845
39	ISO vs ISO	-25.94	13.81	Yes	***	-35.24 to -16.64
40	HSO vs ISO	-11.64	6.193	Yes	*	-20.94 to -2.335
41	HSO vs HSO	-1.757	0.9349	No	ns	-11.06 to 7.545
42	HSO vs ISO	-24.24	12.90	Yes	***	-33.54 to -14.94
43	ISO vs HSO	9.880	5.258	Yes	*	0.5787 to 19.18
44	ISO vs ISO	-12.61	6.709	Yes	**	-21.91 to -3.305
45	HSO vs ISO	-22.49	11.97	Yes	***	-31.79 to -13.19
46						
47						
48						
49						

1way ANOVA of Resultados | Tabular results

ES 09:14 p.m. 31/07/2019

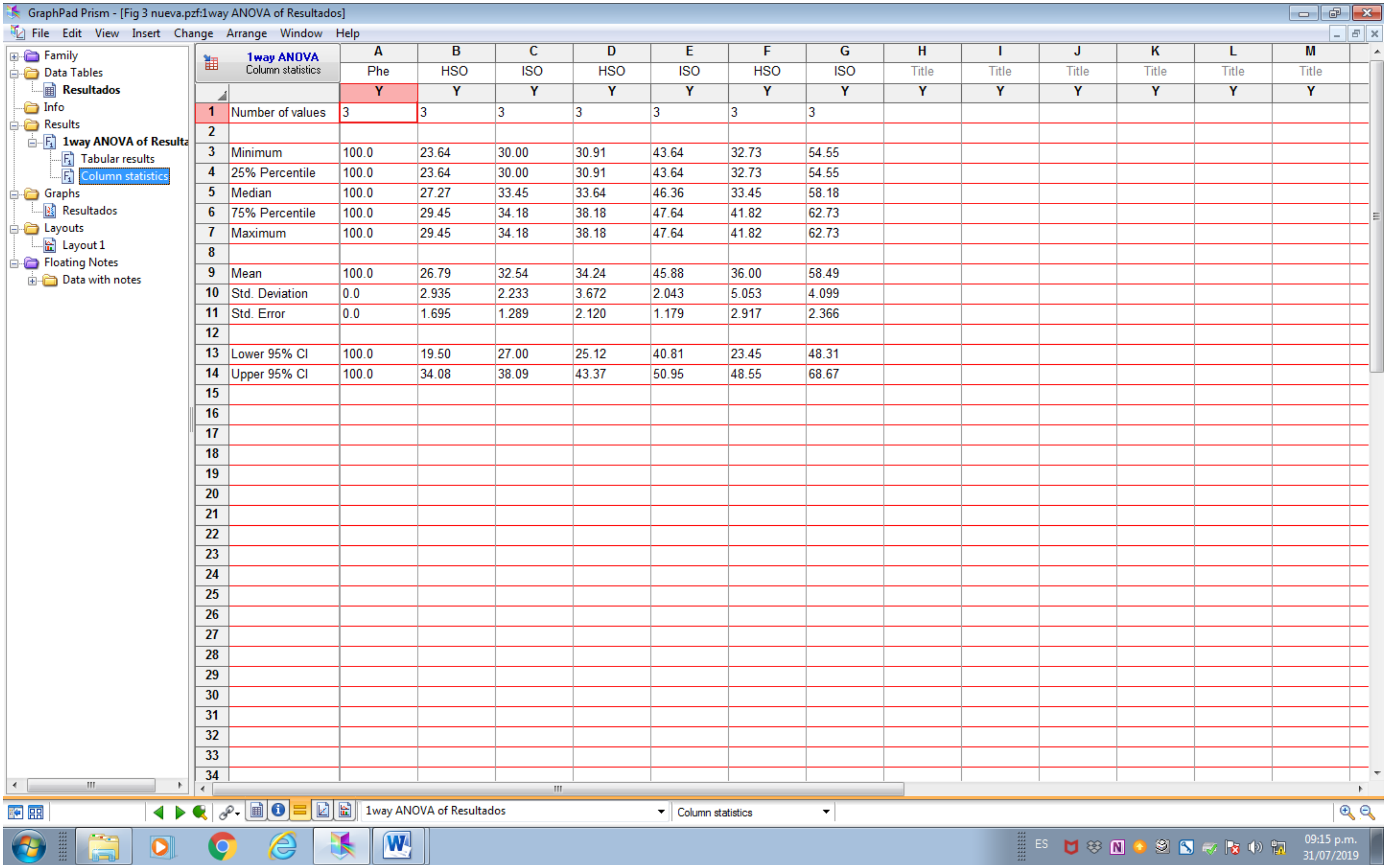

	A	B	C	D	E	F	G
	Phe	HSO	ISO	HSO	ISO	HSO	ISO
	Y	Y	Y	Y	Y	Y	Y
1 Number of values	3	3	3	3	3	3	3
2							
3 Minimum	100.0	23.64	30.00	30.91	43.64	32.73	54.55
4 25% Percentile	100.0	23.64	30.00	30.91	43.64	32.73	54.55
5 Median	100.0	27.27	33.45	33.64	46.36	33.45	58.18
6 75% Percentile	100.0	29.45	34.18	38.18	47.64	41.82	62.73
7 Maximum	100.0	29.45	34.18	38.18	47.64	41.82	62.73
8							
9 Mean	100.0	26.79	32.54	34.24	45.88	36.00	58.49
10 Std. Deviation	0.0	2.935	2.233	3.672	2.043	5.053	4.099
11 Std. Error	0.0	1.695	1.289	2.120	1.179	2.917	2.366
12							
13 Lower 95% CI	100.0	19.50	27.00	25.12	40.81	23.45	48.31
14 Upper 95% CI	100.0	34.08	38.09	43.37	50.95	48.55	68.67

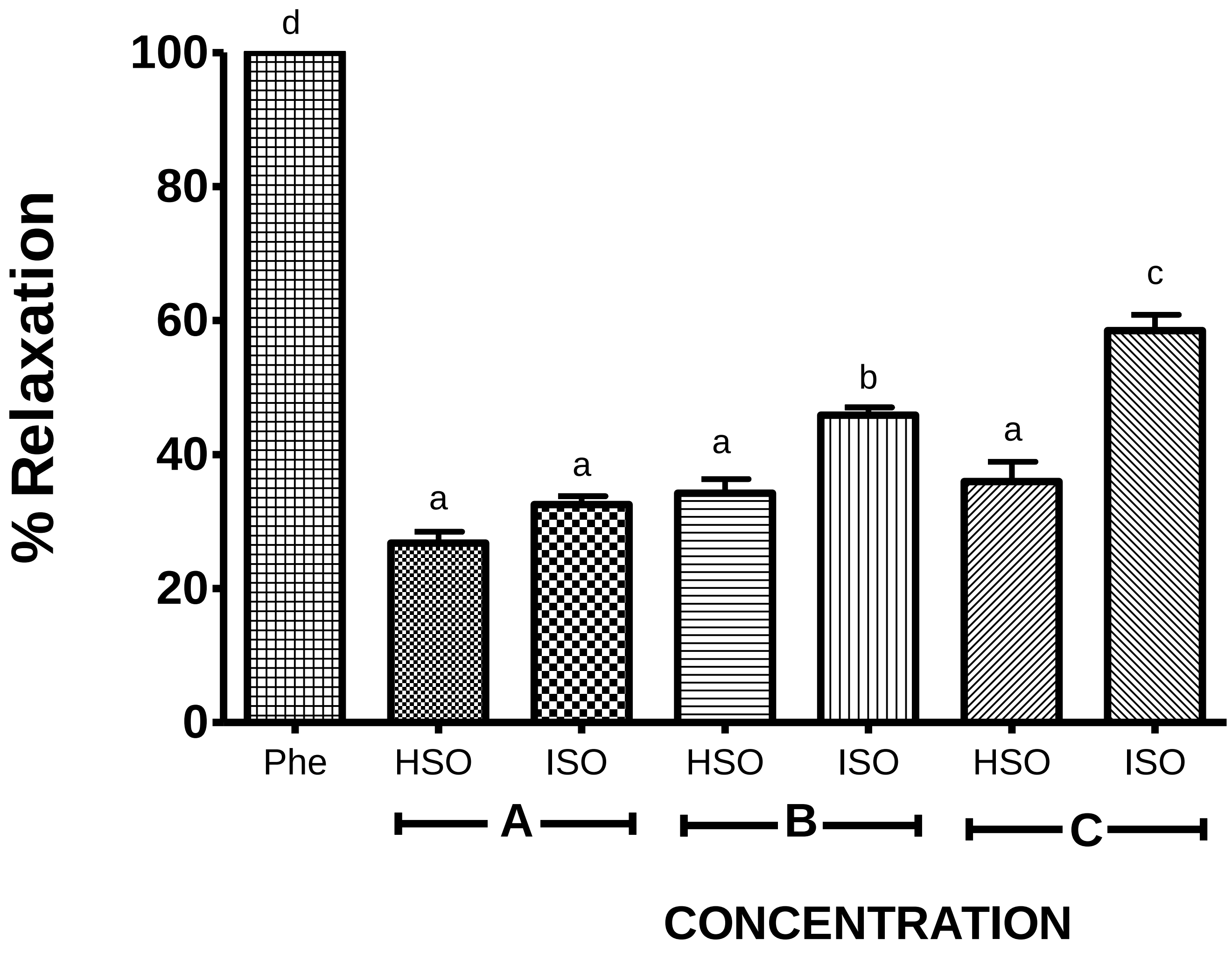
% Relaxation
100
80
60
40
20
0
d
a
a
a
b
a
c
Phe
HSO
ISO
HSO
ISO
HSO
ISO
A
B
C
CONCENTRATION

Role of NO in the effects of HSO and ISO

(Fig 4)

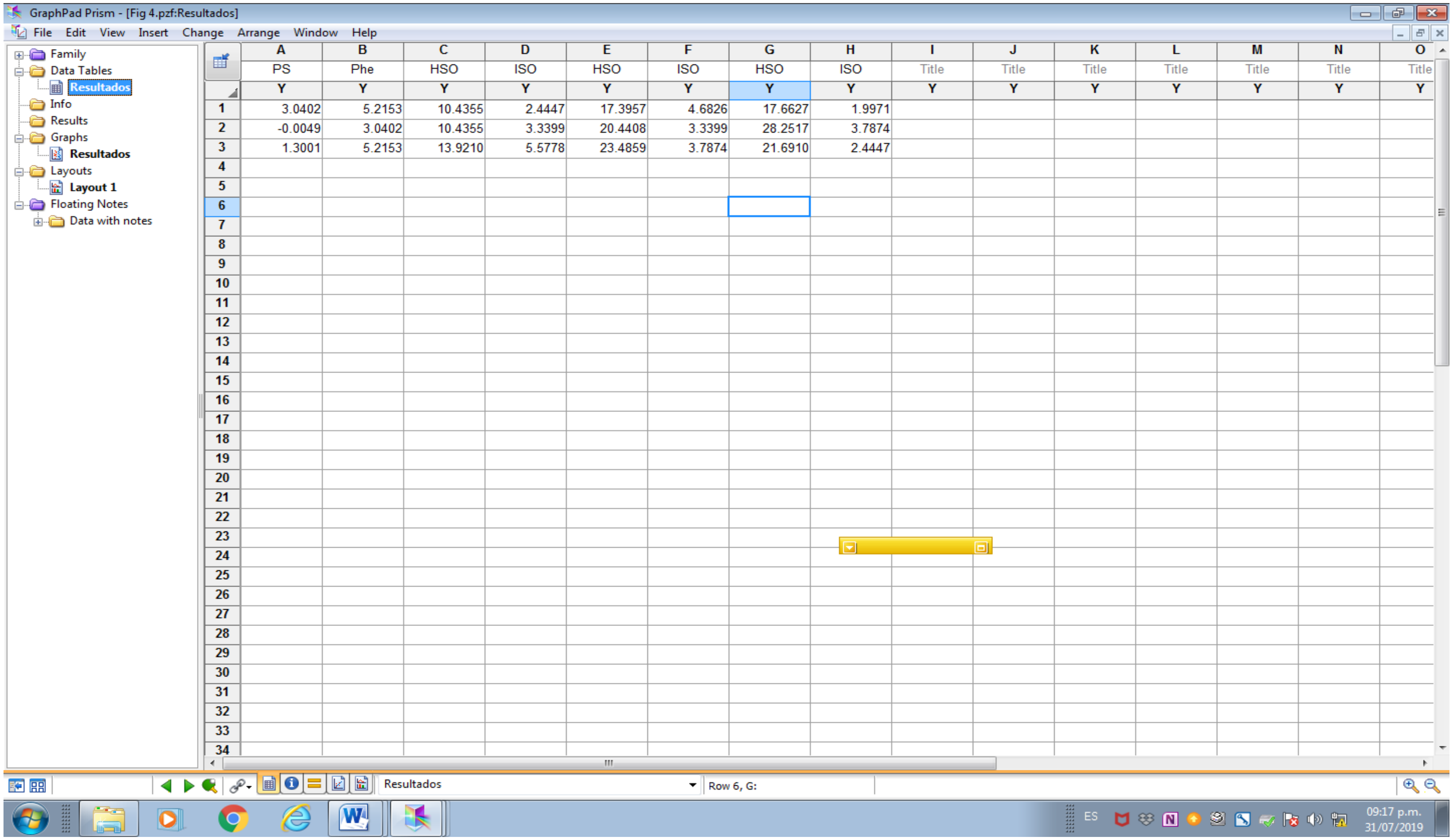

GraphPad Prism - [Fig 4.pzf:Resultados]

	A	B	C	D	E	F	G	H	I	J	K	L	M	N	O
	PS	Phe	HSO	ISO	HSO	ISO	HSO	ISO	Title	Title	Title	Title	Title	Title	Title
	Y	Y	Y	Y	Y	Y	Y	Y	Y	Y	Y	Y	Y	Y	Y
1	3.0402	5.2153	10.4355	2.4447	17.3957	4.6826	17.6627	1.9971							
2	-0.0049	3.0402	10.4355	3.3399	20.4408	3.3399	28.2517	3.7874							
3	1.3001	5.2153	13.9210	5.5778	23.4859	3.7874	21.6910	2.4447							

GraphPad Prism - [Fig 4.pzf:1way ANOVA of Resultados]

1way ANOVA
Tabular results

1	Table Analyzed	Resultados				
2						
3	Repeated Measures ANOVA					
4	P value	< 0.0001				
5	P value summary	***				
6	Are means signif. different? (P < 0.05)	Yes				
7	Number of groups	8				
8	F	34.30				
9	R squared	0.9449				
10						
11	Was the pairing significantly effective?					
12	R squared	0.008756				
13	F	1.122				
14	P value	0.3532				
15	P value summary	ns				
16	Is there significant matching? (P < 0.05)	No				
17						
18	ANOVA Table	SS	df	MS		
19	Treatment (between columns)	1470	7	210.0		
20	Individual (between rows)	13.74	2	6.872		
21	Residual (random)	85.73	14	6.124		
22	Total	1570	23			
23						
24	Tukey's Multiple Comparison Test	Mean Diff.	q	Significant? P < 0.05?	Summary	95% CI of diff
25	PS vs Phe	-3.045	2.131	No	ns	-10.17 to 4.084
26	PS vs HSO	-10.15	7.106	Yes	**	-17.28 to -3.023
27	PS vs ISO	-2.342	1.639	No	ns	-9.472 to 4.787
28	PS vs HSO	-19.00	13.30	Yes	***	-26.13 to -11.87
29	PS vs ISO	-2.492	1.744	No	ns	-9.621 to 4.638
30	PS vs HSO	-21.09	14.76	Yes	***	-28.22 to -13.96
31	PS vs ISO	-1.298	0.9084	No	ns	-8.427 to 5.831
32	Phe vs HSO	-7.107	4.974	No	ns	-14.24 to 0.02235
33	Phe vs ISO	0.7028	0.4919	No	ns	-6.427 to 7.832
34	Phe vs HSO	-15.95	11.16	Yes	***	-23.08 to -8.821

GraphPad Prism - [Fig 4.pzf:1way ANOVA of Resultados]

1way ANOVA
Tabular results

22	Total	1570	23			
23						
24	Tukey's Multiple Comparison Test	Mean Diff.	q	Significant? P < 0.05?	Summary	95% CI of diff
25	PS vs Phe	-3.045	2.131	No	ns	-10.17 to 4.084
26	PS vs HSO	-10.15	7.106	Yes	**	-17.28 to -3.023
27	PS vs ISO	-2.342	1.639	No	ns	-9.472 to 4.787
28	PS vs HSO	-19.00	13.30	Yes	***	-26.13 to -11.87
29	PS vs ISO	-2.492	1.744	No	ns	-9.621 to 4.638
30	PS vs HSO	-21.09	14.76	Yes	***	-28.22 to -13.96
31	PS vs ISO	-1.298	0.9084	No	ns	-8.427 to 5.831
32	Phe vs HSO	-7.107	4.974	No	ns	-14.24 to 0.02235
33	Phe vs ISO	0.7028	0.4919	No	ns	-6.427 to 7.832
34	Phe vs HSO	-15.95	11.16	Yes	***	-23.08 to -8.821
35	Phe vs ISO	0.5536	0.3875	No	ns	-6.576 to 7.683
36	Phe vs HSO	-18.04	12.63	Yes	***	-25.17 to -10.92
37	Phe vs ISO	1.747	1.223	No	ns	-5.382 to 8.877
38	HSO vs ISO	7.810	5.466	Yes	*	0.6805 to 14.94
39	HSO vs HSO	-8.843	6.190	Yes	*	-15.97 to -1.714
40	HSO vs ISO	7.661	5.362	Yes	*	0.5313 to 14.79
41	HSO vs HSO	-10.94	7.656	Yes	**	-18.07 to -3.808
42	HSO vs ISO	8.854	6.197	Yes	*	1.725 to 15.98
43	ISO vs HSO	-16.65	11.66	Yes	***	-23.78 to -9.524
44	ISO vs ISO	-0.1492	0.1044	No	ns	-7.279 to 6.980
45	ISO vs HSO	-18.75	13.12	Yes	***	-25.88 to -11.62
46	ISO vs ISO	1.044	0.7310	No	ns	-6.085 to 8.174
47	HSO vs ISO	16.50	11.55	Yes	***	9.375 to 23.63
48	HSO vs HSO	-2.094	1.466	No	ns	-9.224 to 5.035
49	HSO vs ISO	17.70	12.39	Yes	***	10.57 to 24.83
50	ISO vs HSO	-18.60	13.02	Yes	***	-25.73 to -11.47
51	ISO vs ISO	1.194	0.8354	No	ns	-5.936 to 8.323
52	HSO vs ISO	19.79	13.85	Yes	***	12.66 to 26.92
53						
54						
55						

GraphPad Prism - [Fig 4.pzf:1way ANOVA of Resultados]

1way ANOVA Column statistics

		A	B	C	D	E	F	G	H
		PS	Phe	HSO	ISO	HSO	ISO	HSO	ISO
		Y	Y	Y	Y	Y	Y	Y	Y
1	Number of values	3	3	3	3	3	3	3	3
2									
3	Minimum	-0.0049	3.040	10.44	2.445	17.40	3.340	17.66	1.997
4	25% Percentile	-0.0049	3.040	10.44	2.445	17.40	3.340	17.66	1.997
5	Median	1.300	5.215	10.44	3.340	20.44	3.787	21.69	2.445
6	75% Percentile	3.040	5.215	13.92	5.578	23.49	4.683	28.25	3.787
7	Maximum	3.040	5.215	13.92	5.578	23.49	4.683	28.25	3.787
8									
9	Mean	1.445	4.490	11.60	3.787	20.44	3.937	22.54	2.743
10	Std. Deviation	1.528	1.256	2.012	1.614	3.045	0.6837	5.345	0.9317
11	Std. Error	0.8820	0.7250	1.162	0.9317	1.758	0.3947	3.086	0.5379
12									
13	Lower 95% CI	-2.350	1.371	6.598	-0.2214	12.88	2.238	9.258	0.4286
14	Upper 95% CI	5.240	7.610	16.60	7.796	28.01	5.635	35.81	5.058

1way ANOVA of Resultados · Column statistics

09:22 p.m. 31/07/2019

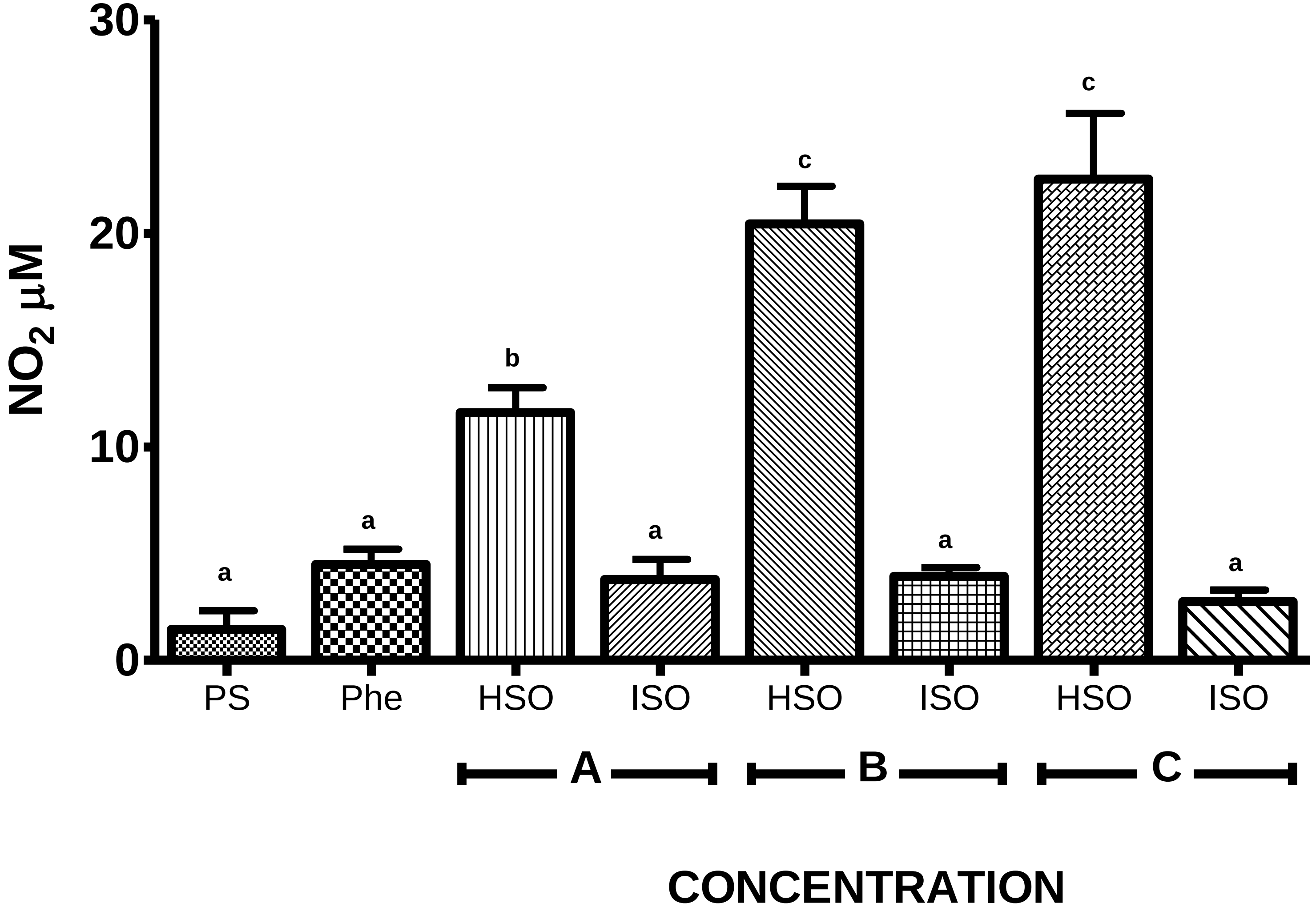

NO2 µM
30
20
10
0
a
a
b
a
c
a
c
a
PS
Phe
HSO
ISO
HSO
ISO
HSO
ISO
A
B
C
CONCENTRATION

Fig 5

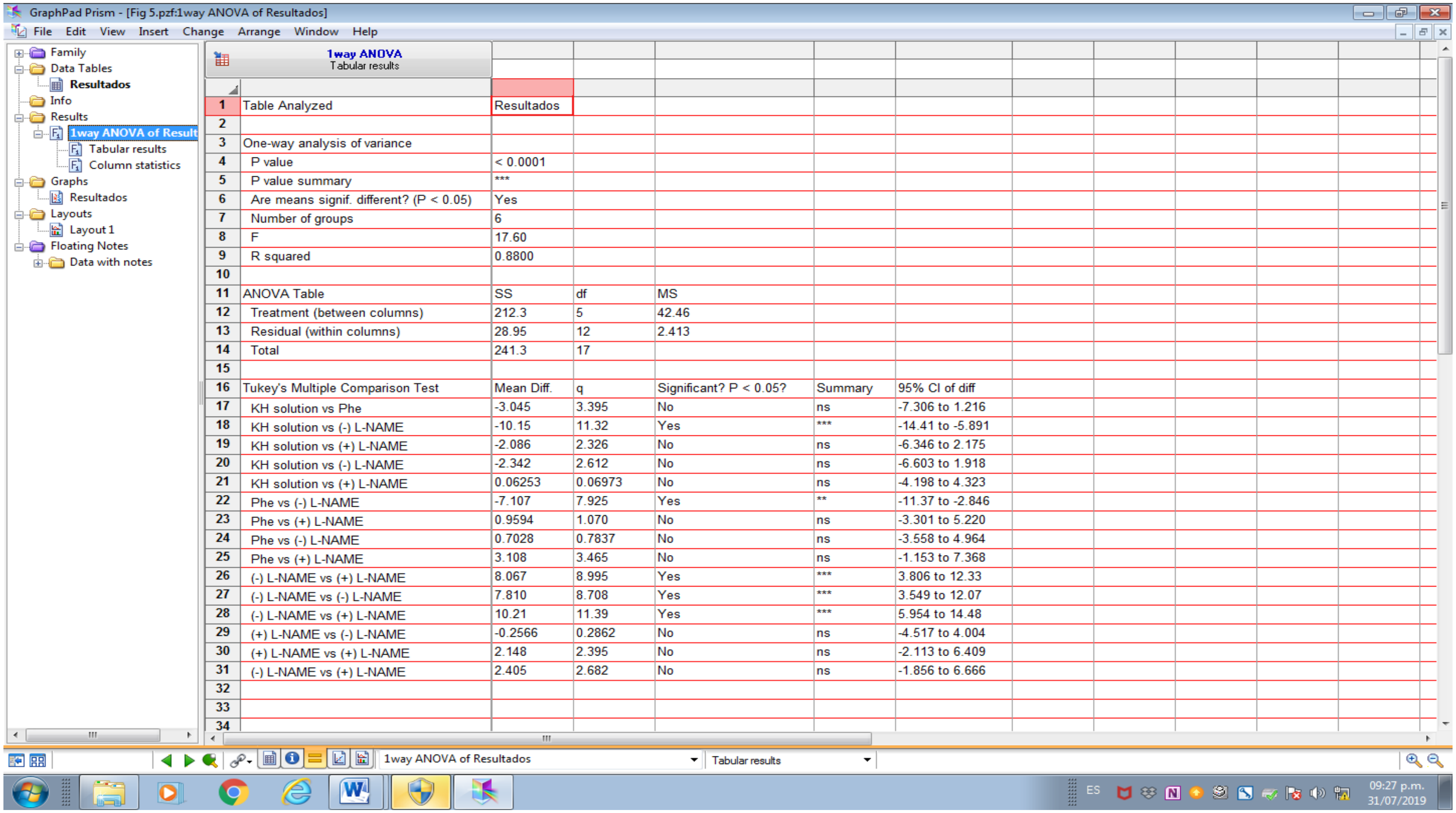

Row						
1	Table Analyzed	Resultados				
2						
3	One-way analysis of variance					
4	P value	< 0.0001				
5	P value summary	***				
6	Are means signif. different? (P < 0.05)	Yes				
7	Number of groups	6				
8	F	17.60				
9	R squared	0.8800				
10						
11	ANOVA Table	SS	df	MS		
12	Treatment (between columns)	212.3	5	42.46		
13	Residual (within columns)	28.95	12	2.413		
14	Total	241.3	17			
15						
16	Tukey's Multiple Comparison Test	Mean Diff.	q	Significant? P < 0.05?	Summary	95% CI of diff
17	KH solution vs Phe	-3.045	3.395	No	ns	-7.306 to 1.216
18	KH solution vs (-) L-NAME	-10.15	11.32	Yes	***	-14.41 to -5.891
19	KH solution vs (+) L-NAME	-2.086	2.326	No	ns	-6.346 to 2.175
20	KH solution vs (-) L-NAME	-2.342	2.612	No	ns	-6.603 to 1.918
21	KH solution vs (+) L-NAME	0.06253	0.06973	No	ns	-4.198 to 4.323
22	Phe vs (-) L-NAME	-7.107	7.925	Yes	**	-11.37 to -2.846
23	Phe vs (+) L-NAME	0.9594	1.070	No	ns	-3.301 to 5.220
24	Phe vs (-) L-NAME	0.7028	0.7837	No	ns	-3.558 to 4.964
25	Phe vs (+) L-NAME	3.108	3.465	No	ns	-1.153 to 7.368
26	(-) L-NAME vs (+) L-NAME	8.067	8.995	Yes	***	3.806 to 12.33
27	(-) L-NAME vs (-) L-NAME	7.810	8.708	Yes	***	3.549 to 12.07
28	(-) L-NAME vs (+) L-NAME	10.21	11.39	Yes	***	5.954 to 14.48
29	(+) L-NAME vs (-) L-NAME	-0.2566	0.2862	No	ns	-4.517 to 4.004
30	(+) L-NAME vs (+) L-NAME	2.148	2.395	No	ns	-2.113 to 6.409
31	(-) L-NAME vs (+) L-NAME	2.405	2.682	No	ns	-1.856 to 6.666
32						
33						
34						

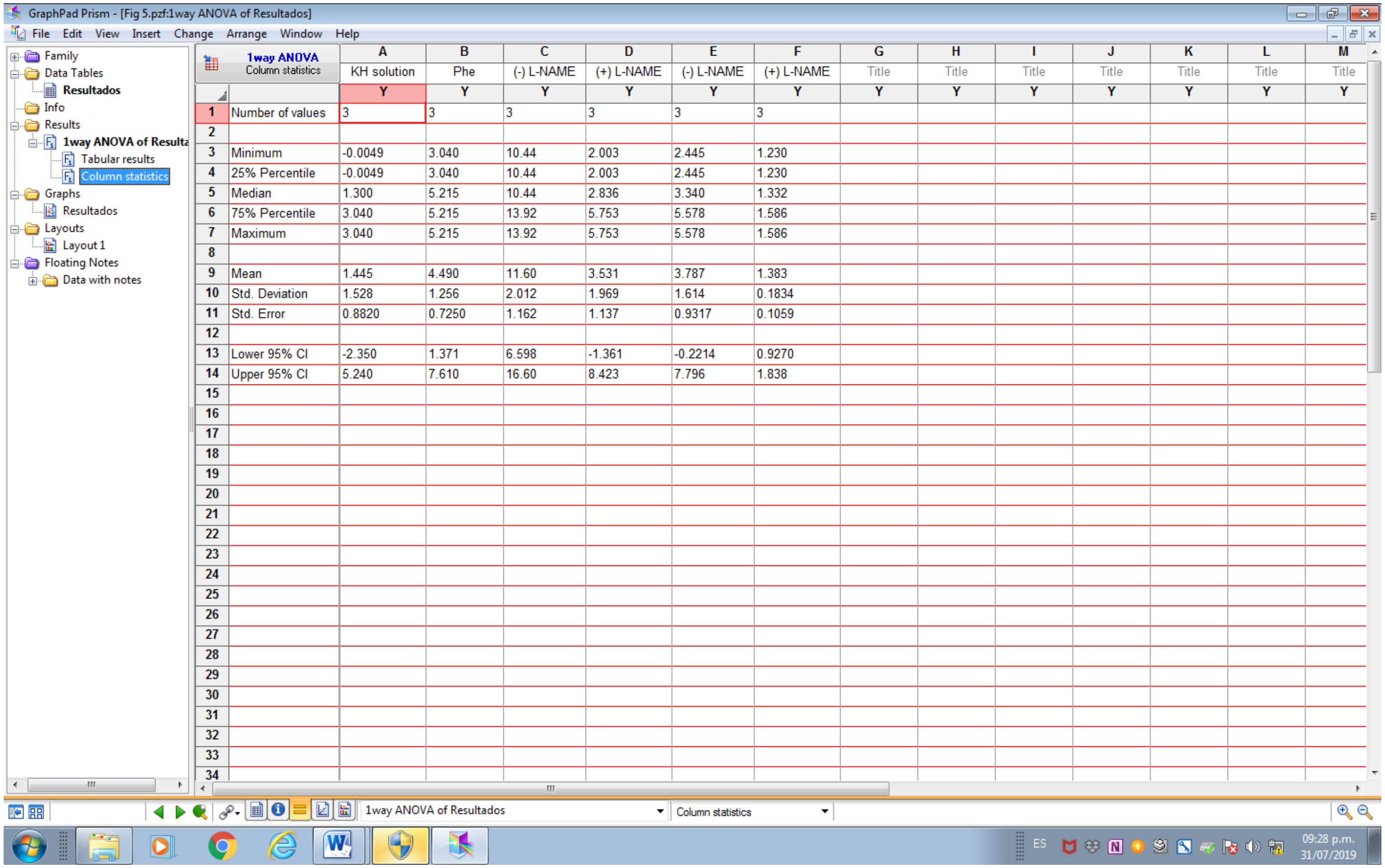

1way ANOVA Column statistics	A KH solution	B Phe	C (-) L-NAME	D (+) L-NAME	E (-) L-NAME	F (+) L-NAME
	Y	Y	Y	Y	Y	Y
1 Number of values	3	3	3	3	3	3
2						
3 Minimum	-0.0049	3.040	10.44	2.003	2.445	1.230
4 25% Percentile	-0.0049	3.040	10.44	2.003	2.445	1.230
5 Median	1.300	5.215	10.44	2.836	3.340	1.332
6 75% Percentile	3.040	5.215	13.92	5.753	5.578	1.586
7 Maximum	3.040	5.215	13.92	5.753	5.578	1.586
8						
9 Mean	1.445	4.490	11.60	3.531	3.787	1.383
10 Std. Deviation	1.528	1.256	2.012	1.969	1.614	0.1834
11 Std. Error	0.8820	0.7250	1.162	1.137	0.9317	0.1059
12						
13 Lower 95% CI	-2.350	1.371	6.598	-1.361	-0.2214	0.9270
14 Upper 95% CI	5.240	7.610	16.60	8.423	7.796	1.838

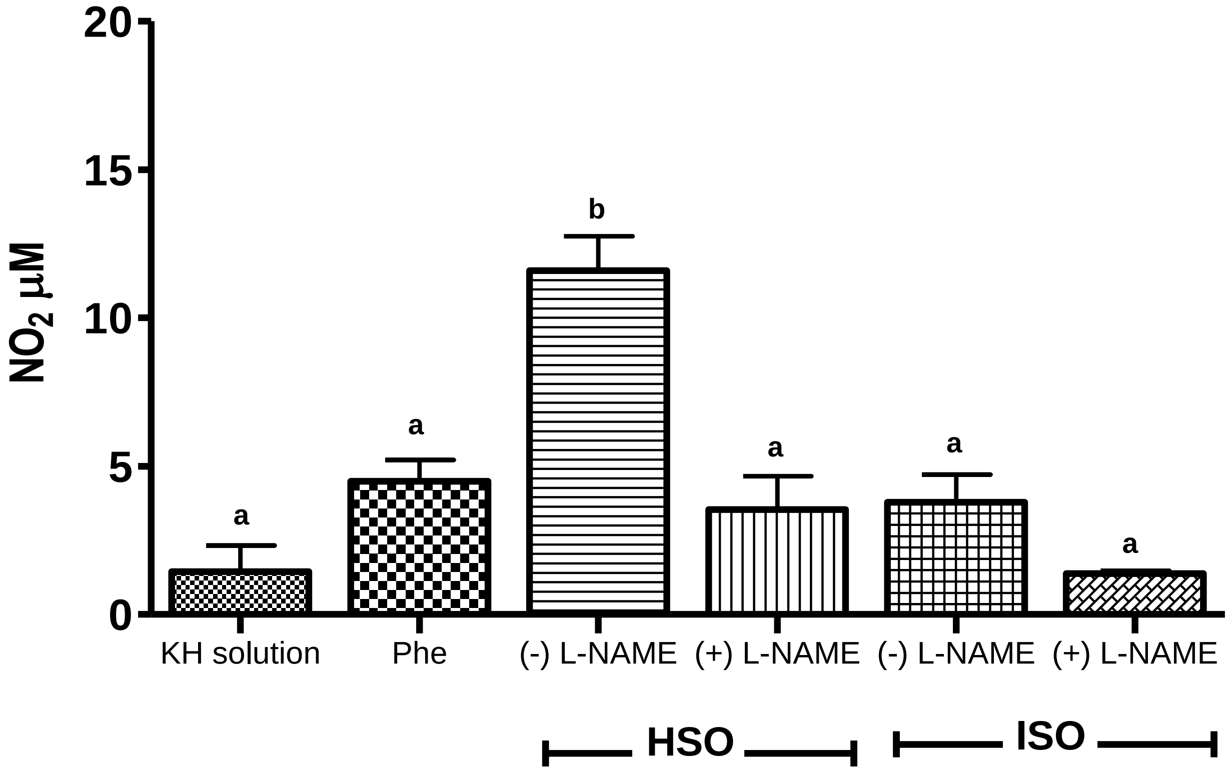

20
15
10
5
0
NO_2 µM
b
a
a
a
a
a
KH solution
Phe
(-) L-NAME
(+) L-NAME
(-) L-NAME
(+) L-NAME
HSO
ISO
