# Supplementary material for: Effect of the double bond conjugation on the vascular physiology and nitric oxide production of isomers of eicosapentaenoic and docosahexaenoic acids prepared from shark oil
Source: PLoS One. 2020 Feb 27;15(2):e0229435. doi: 10.1371/journal.pone.0229435 (PMC7046235; doi:10.1371/journal.pone.0229435)
Supplement: S1 Text — (DOCX) [file pone.0229435.s002.docx]

**EFFECT OF LOWER CONCENTRATION OF N-3 PUFA ON THE VASCULAR TONE.**

**Effect of ISO and HSO at concentrations close to 100 µM of EPA and DHA isomers**

To evaluate whether EPA and DHA exert similar effects upon vascular tone than higher concentrations (as in this study); recently, we decided to test lower concentrations (in the level of 100 µM) in a new study. In mixture form as in HSO (hydrolyzed shark oil) or ISO (isomerized shark oil) and pure forms of EPA, DHA each one in a pure form and mixtures between pure forms (i.e., mixtures of DHA + EPA), as non-conjugated or conjugated forms.

First, we tested conjugated-PUFA, CEPA, and CDHA, (i.e., ISO) or non-conjugated PUFA, EPA and DHA, (i.e. HSO) in mixtures with other fatty acids present in shark oil, using close to 100 µM of each of these PUFA, in the presence of Phe 2µM, which precontracted the vessels. We observed that conjugated-PUFA (HSO) (Figure 1B and Figure 1C) induced a higher relaxation than the non-conjugated form (Fig 1A and Figure 1C) in the presence of endothelium (+E). Both kinds of treatments responded to the vasodilator agent (ACh). That indicates similar results than the observed at higher concentrations of PUFA (A, B y C).


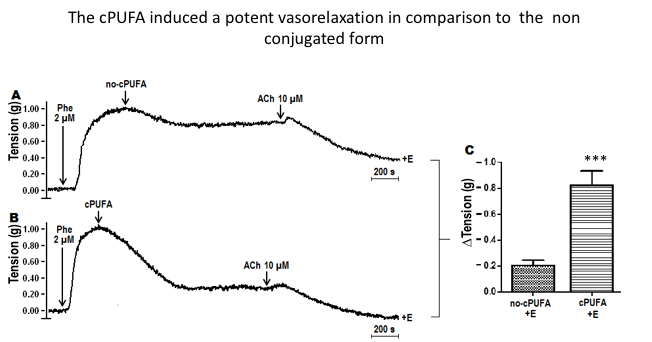


Figure 1. The recordings represent the vascular response of isolated rat aortic rings and pre-contracted (2 μM phenylephrine, Phe) and treated in the presence of A) non-conjugated PUFA (DHA and EPA among other FA) at about 100 µM each, and B) conjugated PUFA (CDHA and CEPA among other FA) at about 100 µM each. In C), there is a decrease in tension (∆Tension - proportional to vasorelaxation) in grams for each treatment. + E, presence of endothelium. Mean ± SD. n = 3, p <0.001, cPUFA *vs* none-cPUFA (***). Acetylcholine (ACh) represents the positive control of vasorelaxation.

Moreover, similar to the results observed at the higher concentration used in the present work, the vasodilation induced by conjugated PUFA (ISO) was independent of NO.

Thus, the rat aortic vessels in the presence of endothelium were subsequently precontracted with 2 µM Phe, followed by a single administration of conjugated PUFA or non-conjugated PUFA at about 100 µM, and in the presence or absence of a 30 min pretreatment of L-NAME 100 µM or Indomethacin 40 µM. Regarding the procedures, the functionality of the endothelium was evaluated via the amount of relaxation induced by ACh 10 μM. Sustained relaxation in response to ACh confirmed the presence of a functional endothelium.

This effect was independent of the derivatives of arachidonic acid (Figure 2) because of the preincubation of L-NAME or Indomethacin as a blocker of arachidonic derivatives were not capable of inhibiting the vasodilator effect induced by conjugated PUFA.


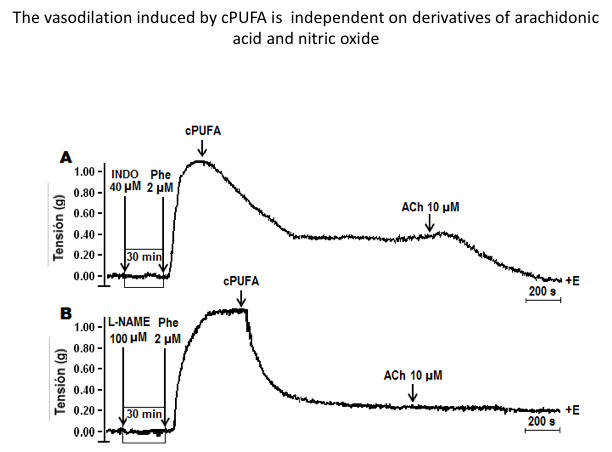


Figure 2. The recordings represent the effect induced by cPUFA on aortic rings pre-incubated with A) indomethacin (INDO) 40 µM and B) L-NAME 100 µM. + E, presence of endothelium.

The effect was validated with the addition of ACh as control of relaxation in the presence of endothelium (E+); such effect was inhibited in the presence of L-NAME, but not in the presence of indomethacin.

**Effect of EPA and DHA isomers alone and in mixtures, at lower concentrations.**

Additionally, in the new study, we preliminarily evaluated the % relaxation of samples of pure standards of EPA and DHA in the conjugated and non-conjugated forms, alone and combined (EPA and DHA) and compared them with the % relaxation induced by the systems of this study (i.e. ISO and HSO at concentrations A, B and C of DHA and EPA isomers) (Figure 3).

Figure 3. Preliminary results of relaxation activity exerted by pure standards of EPA and DHA in the conjugated and non-conjugated forms, alone and combined, compared to the relaxation activity of ISO and HSO samples at the different concentrations (A, B and C). All treatments with pure standards were done once. Thus, results can only show trends.

Data resulted from preliminary experiments of such work (Figure 3), can be summarized, in the context of this study, as follow. 1) All pure standard samples exerted a relaxation effect, which can be as intense as that of ISO and HSO mixtures. 2) Conjugation of individual standards seems no to have the same impact on the relaxation activity of pure samples of DHA and EPA as samples of HSO and ISO. 3) EPA alone seems to induce higher relaxation than DHA. And 4) when DHA and EPA are combined, they seem to exert a synergist effect. Except for what is mentioned in point one, the other points generate an interesting surprise. It allows inferring that each FA (EPA or DHA) shows different effect; and that others FA present in HSO and ISO induce a critical impact on the vascular tone. Interestingly, the global impact of the mixtures (i.e., HSO and ISO) was of relaxation, which is favorable. It evidences that the influence of EPA and DHA was predominant over the impact of the others FA. However, the effect of the others FA should not be ignored and also should be studied.
